# Evaluation of Sox2 binding affinities for distinct DNA patterns using steered molecular dynamics simulation

**DOI:** 10.1002/2211-5463.12316

**Published:** 2017-10-09

**Authors:** Dhanusha Yesudhas, Muhammad Ayaz Anwar, Suresh Panneerselvam, Han‐Kyul Kim, Sangdun Choi

**Affiliations:** ^1^ Department of Molecular Science and Technology Ajou University Suwon Korea

**Keywords:** molecular dynamics, protein–DNA interaction, Sox2, umbrella sampling

## Abstract

Transcription factors (TFs) are gene expression regulators that bind to DNA in a sequence‐specific manner and determine the functional characteristics of the gene. It is worthwhile to study the unique characteristics of such specific TF‐binding pattern in DNA. Sox2 recognizes a 6‐ to 7‐base pair consensus DNA sequence; the central four bases of the binding site are highly conserved, whereas the two to three flanking bases are variable. Here, we attempted to analyze the binding affinity and specificity of the Sox2 protein for distinct DNA sequence patterns via steered molecular dynamics, in which a pulling force is employed to dissociate Sox2 from Sox2–DNA during simulation to study the behavior of a complex under nonequilibrium conditions. The simulation results revealed that the first two stacking bases of the binding pattern have an exclusive impact on the binding affinity, with the corresponding mutant complexes showing greater binding and longer dissociation time than the experimental complexes do. In contrast, mutation of the conserved bases tends to reduce the affinity, and mutation of the complete conserved region disrupts the binding. It might pave the way to identify the most likely binding pattern recognized by Sox2 based on the affinity of each configuration. The α2‐helix of Sox2 was found to be the key player in the Sox2–DNA association. The characterization of Sox2's binding patterns for the target genes in the genome helps in understanding of its regulatory functions.

**Abbreviations**
bp: base pair
COM: center of mass
Dppa4: developmental pluripotency‐associated 4
emboss: European Molecular Biology Open Software Suite
EMSA: electrophoretic mobility shift assay
ESC: embryonic stem cell
Fgf4: fibroblast growth factor 4
*FHIT*: fragile histidine triad
HMG: high‐mobility group box
MD: molecular dynamics
MINT: Motif Identifier for Nucleic acids Trajectory
ns: nanosecond
Oct4: octamer‐binding protein 3/4
PMF: potential of mean force
ps: picosecond
PWM: position weight matrix
RDF: radial distribution function
Rg: reaction coordinate
SMD: steered molecular dynamics
Sox2: SRY (sex‐determining region Y)‐box 2
SRY: sex‐determining region Y
TF: transcription factor
TSS: transcription start site

Sex‐determining region Y (SRY)‐box 2 (Sox2) is the key inducer of stem cell pluripotency along with octamer‐binding protein 3/4 (Oct4), Nanog, and Krüppel‐like factor 4. A Sox2 protein interacts with DNA through its high‐mobility group box (HMG) domain, which consists of 79 amino acid residues 1. HMG domains are unique because they interact with the minor groove of the DNA helix and induce a drastic bend in the DNA molecule. DNA bending regulates the assembly of higher‐order DNA–multiprotein (Oct4/Sox2) complexes by facilitating the distal DNA regions and proteins to interact with each other. However, in a cellular context, the tissue‐specific genes require a unique set of distal enhancer element in order to import the cis‐acting regulators. Sox2 chosen to bind a distinct set of promoters in embryonic stem cell (ESCs) and neural stem cells and suggest that additional factors may contribute to the target specificity 2. Sox2 proteins are highly sequence specific, recognizing 6‐ to 7‐base pair (bp) (CTTTGTC) DNA sequences 3. The core motif (TTGT) is the preferred binding site for all 20 Sox proteins with the slight allowable variations in the flanking bps 4. Along with the highest affinity consensus sequence, the low and medium binding sites will also determine the binding profile of a transcription factor (TF). Therefore, all exposed genomic elements compete to search for its functional binding site. Thus, predicting the binding site probability from the sequence alone would be desirable to quantify the binding energies of all kmer/TF interactions 4.

Precise control of gene expression relies on the ability of TFs to recognize a specific pattern at the DNA binding sites. The process of selection of an enhancer by a TF in a combinatorial fashion to regulate gene expression remains only superficially understood 5. One of the reasons for the difficulty of identification of a target binding pattern in DNA is the variability of the binding sites for a single TF, and the nature of the allowable variations is not well understood either 6. Nevertheless, identification of a binding site pattern in DNA for the factors involved in expression regulation is a necessary first step in determining which factors regulate the gene and how. Some researchers have attempted to determine the recognition code that determines the specific DNA bp likely to bind to the precise amino acids, in the context of a particular structural class of DNA‐binding proteins. These approaches are developed from the databases of well‐defined protein–DNA interactions 5, 7, 8 from computer modeling 9, or from experiments based on *in vitro* selection from a randomized library 10.

The position weight matrix (PWM) is a frequently used method for prediction of a binding pattern for TFs 11. Even though there are difficulties inherent in the use of a PWM, it is an appropriate representation to identify the candidate sites for TFs 5, 12. On the other hand, there is a chance for some bps to show more than fivefold deviation (expected/observed). Such pairs are identified in several structural TF families, including the Sox family proteins 13, 14. Thus, to enhance the accuracy, it is necessary to analyze the TF‐binding patterns that are obtained from these databases.

High‐mobility group box protein like Sox2 has the capability to bind and activate other TFs with and without the presence of DNA. These DNA binding domain proteins are highly flexible or partially unfolded state and become folded when it bound to its target site. Therefore, measuring the equilibrium energetics of this HMG domain with its DNA is necessary to identify the best binding site pattern for the protein. Understanding the force driving the protein–DNA complex formation involves the measurement of Gibbs free energy (electrostatic and nonelectrostatic) and enthalpy–entropy contributions 4, 15. Steered molecular dynamics [(SMD) employs a pulling force to cause a change in structure during simulation to study the behavior of the complex under nonequilibrium conditions] simulation along with umbrella sampling [potential of mean force (PMF)] provide qualitative and quantitative predictions of protein–DNA binding energies of these constructive and nonconstructive sites of Sox2 complexes 14. The DNA binding site for a Sox2 consists of seven bp that includes both conserved and nonconserved regions. The central four bp are highly conserved, whereas the flanking region bps are nonconserved. In the present study, our aim was to analyze and characterize the impact on the affinity of distinct Sox2‐binding patterns of DNA obtained by mutating the conserved and nonconserved regions of its consensus binding pattern (five mutant patterns were considered for the present study) (Fig. 1 and Fig. S1). Because the conserved and nonconserved bps of Sox2 binding patterns are mutated, this analysis can provide valuable data about the contribution of every bp in the binding pattern toward its binding affinity.

**Figure 1:**
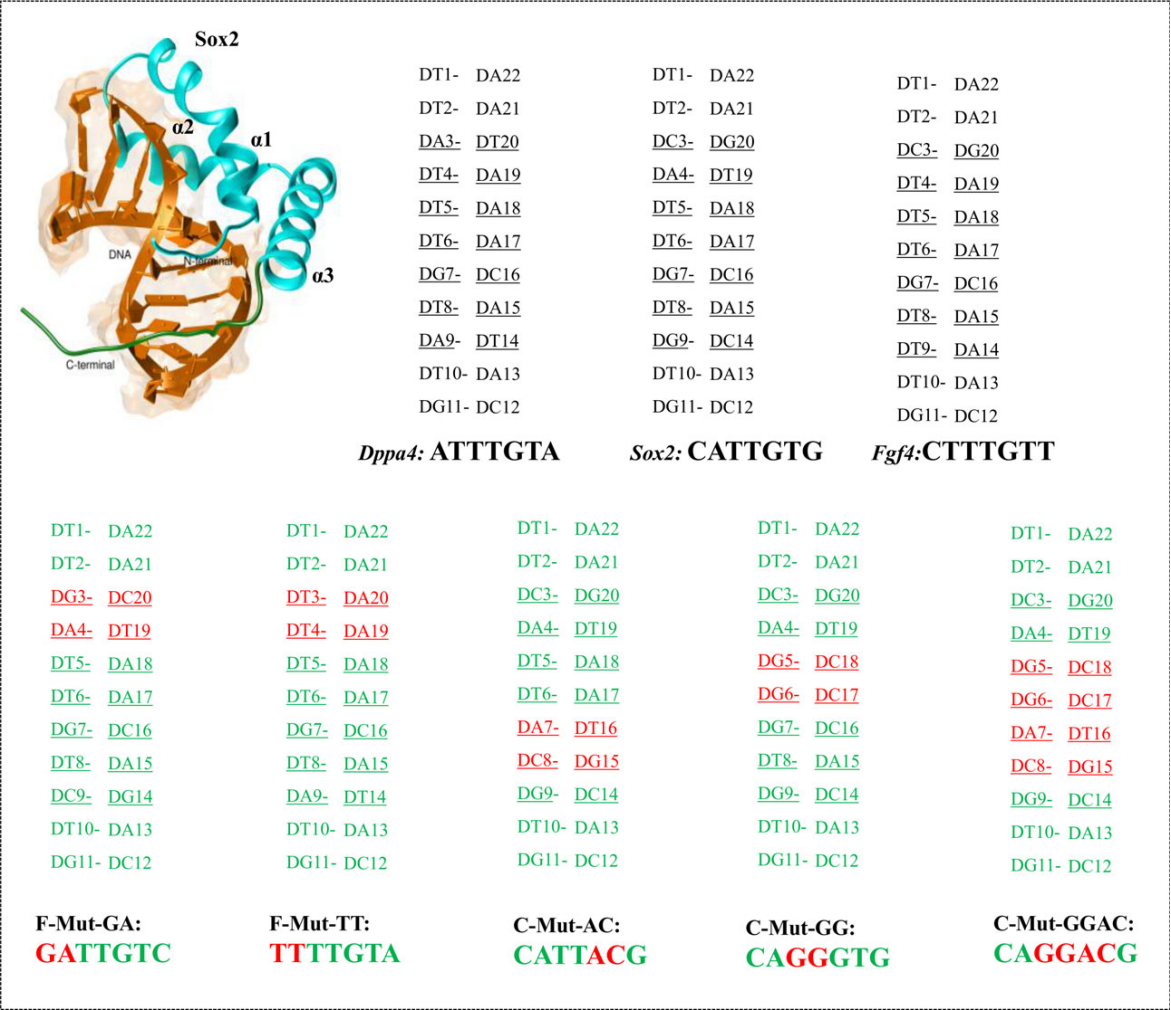
Representation of bp positions for Sox2 binding patterns. The distinct DNA binding patterns for Sox2 protein obtained from Discovery Studio with bp positions numbered. The orange represents the DNA strands with its associated Sox2 indicated in cyan. The bp positions of positive control and mutant complexes are mentioned in black and green, respectively, with mutated bps positions marked in red.

The combinatorial DNA sequence variation and the structural transition of a TF have entered the realm of simulation methods. The altered or mutated DNA patterns [F‐Mut‐GA (GATTGTC), C‐Mut‐AC (CATTACG), C‐Mut‐GG (CAGGGTG), C‐Mut‐GGAC (CAGGACG), and F‐Mut‐TT (TTTTGTA)] along with the experimentally reported DNA patterns [*Dppa4* (ATTTGTA), *Fgf4* (CTTTGTT), and *Sox2* (CATTGTG)] 16 for Sox2 are used here in the analysis by means of a SMD simulation and umbrella sampling. Furthermore, a real‐time qualitative and quantitative analysis of the binding affinity in the complexes by electrophoretic mobility shift assay (EMSA) verifies dependence of this affinity on the binding patterns in DNA.

Finally, it would be more interesting to identify the target genes in the genome that match with various binding patterns of Sox2, thereby assuming its binding specificity. Boyer *et al*. 17 proved that ESCs contain ~ 3000 target genes for TFs Oct4, Sox2, and Nanog. Short TF‐binding motifs are more common in the genome, and the specific uniqueness of gene expression depends on close cooperative binding of several TFs 18, 19. In our study, we explored possible mutant binding patterns for Sox2 along with the adjacent Oct4‐binding sites in human and mouse ESC‐related genes. The identification of a feasible binding pattern for TFs will provide a better insight into the selectivity of its regulation of transcription.

## Results

Protein–DNA interactions are necessary in many biological processes, including DNA transcription, DNA duplication, and gene regulation. Therefore, there is broad interest among researchers in elucidation of the DNA recognition processes in atomic detail. In this study, we used computational strategies to understand the significance of Sox2 binding affinity of experimentally validated binding patterns (*Dppa4*,* Fgf4*, and *Sox2*) 16, and the impacts in the mutation of conserved and nonconserved region bps (F‐Mut‐GA, C‐Mut‐AC, C‐Mut‐GG, C‐Mut‐GGAC, and F‐Mut‐TT) (Fig. 1 and Fig. S1). The flanking (nonconserved) region mutant complexes are termed as F‐Mut derivatives, while the conserved region mutant complexes are termed as C‐Mut derivatives. Based on umbrella sampling (PMF calculation), it is possible to identify the most likely binding pattern recognized by Sox2 and the affinity of each configuration. Thus, we can draw a conclusion about the Sox2 binding specificity based on its binding affinity.

### The dissociation pathway of Sox2 and conformational changes

Sox2 was regarded as a pulling group (the atoms upon which force is applied to dissociate from complex), and DNA served as the immobile reference in SMD analysis. The disassembly pathway for Sox2–DNA complexes comprises major dissociation from the DNA at an early stage of simulation, followed by gradual separation of N‐terminal and C‐terminal loops from the DNA. The experimentally validated complexes (positive control complexes) 16 also followed a similar dissociation pathway: The dissociation started at the interface between the α2‐helix and DNA backbone, followed by α1‐helix detachment because it was interacting with α2, and finally, the α3‐helix dissociated from the DNA. Because the C‐terminal loop was wrapped around the DNA, it required more time for dissociation. Although the dissociation pathway was similar among these positive control complexes, the time and force required for dissociation were different among those complexes, as shown in Table 1.

**Table 1:** Comparison of dissociation time, force, PMF, and the interactions restrained during dissociation of the complexes

Complex	Dissociation time (ps)	Force, kcal·(mol·nm) ^−1^	PMF, kcal·mol^−1^	Major interactions restrained during dissociation	C‐terminal interactions restrained during dissociation
*Dppa4*	180	2000	126.815	Arg15–DA19, Arg19–DT20, Ser34–DT4, Lys35–DT4	Arg2–DA17, Lys4–DA18, Arg5–DT8, Arg5–DA9, Lys71–DT10, Arg75–DG11, Lys79–DA15
*Fgf4*	162	2150	113.65	Arg2–DA17, Arg5–DT9, Asn8–DT6, Ser31–DT4, Ser34–DT4, Lys35–DT5, Lys71–DT10, Tyr72–DC16, Arg75–DC16	Arg73‐DG11, Arg75‐DC16, Arg76‐DA15, Lys77‐DC16
*Sox2*	160	1800	103.151	Lys4–DC17, Arg5–DC8, Phe10–DA7, Arg19–DT19, Asn30–DG20, Ser31–D21	Lys71–DT10, Tyr72–DA15, Arg76–DC14, Arg76–DT10, Lys77–DC16, Thr80–DT1
F‐Mut‐GA	190	1750	124.78	Arg2–DA17, Arg5–DC16, Asn8–DT6, Phe10–DT5Ser34–DT5, Trp41–DT6	Arg2–DC16, Arg2–DA17, Lys71–DT10, Arg75–DG11, Arg76–DA15, Lys77–DT2, Thr80–DT1
C‐Mut‐AC	150	1650	98.518	Asn8–DT6, Arg18–DT5, Ser31–DC3, Ser31–DA4, Ser34–DT5, Trp41–DA7	Arg5–DC8, Trp41–DA7, Tyr72–DT16, Arg73–DG11, Lys77–DA17
C‐Mut‐GG	150	1600	87.46	Arg5–DT8, Arg5–DT8, Arg5–DG9, Trp41–DG7, Lys42–DG6, Lys71‐DT10	Tyr70–DG9, Tyr72–DC16, Arg75–DC16, Lys77–DC16, Thr80–DT2
C‐Mut‐GGAC	180	2100	46.008	Arg5–DC8, Asn5–DC8, Asn5–DA7, Trp41–DG6	Lys71–DT10, Arg75–DT10, Arg75–DG11, Thr80–DT1, Thr80–DT2
F‐Mut‐TT	200	2000	123.7	Arg2–DA17, Arg5–DT8, Arg5–DA9, Arg5–DC16, Arg15–DA18, Arg15–DA19, Arg15–DA20, Trp41–DT6	Lys4–DA18, Arg60–DA9, Tyr72–DA18, Arg76–DT8, Arg76–DA9

**Figure 2:**
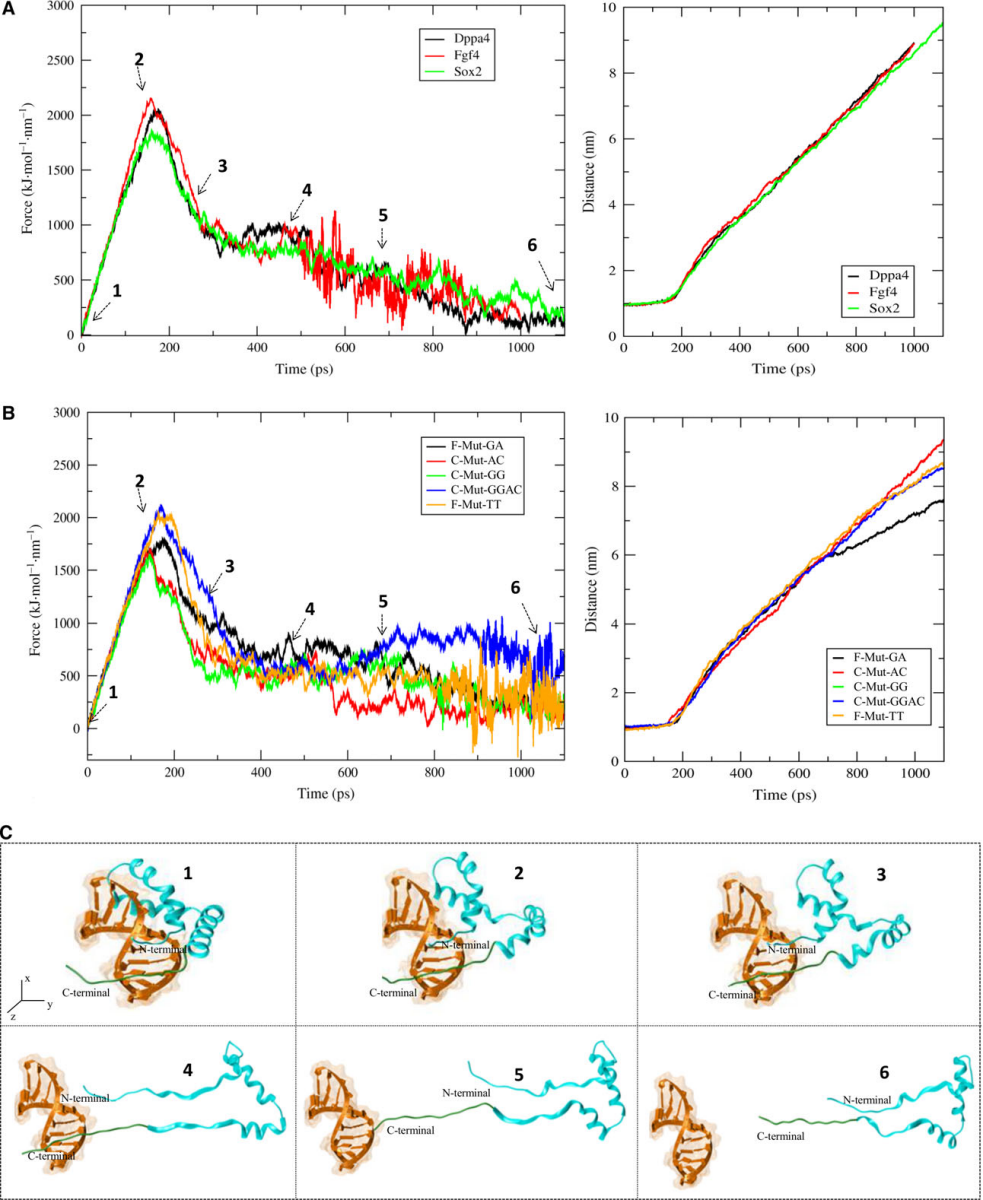
The dissociation pathway along with structural deformation during SMD analysis. (A) The dissociation pathway of Sox2 in the positive control complexes, showing the time and force required for dissociation. The increase in the distances between the protein and DNA during dissociation is also shown. (B) The dissociation pathway of Sox2 in complex with a mutant sequence showing the time and force required for dissociation. The increase in the distances between the protein and DNA during dissociation is also indicated. (C) General representation of the dissociation process showing a Sox2 dissociated from DNA and the distance at different stages of separation.

We found that the dissociation of Sox2 from the *Sox2* promoter was quicker than that from *Fgf4* and *Dppa4* promoters. For *Sox2* promoter complex, the major dissociation started at 160 picosecond (ps), and the complete N‐terminal dissociation occurred at 400 ps, thereby restraining the interaction of the N terminus with DA17 (the labels are defined in Fig. 1). Nonetheless, the C‐terminal loop dissociation was more complicated due to the strong interaction of Tyr72 and Arg76 with DA15 and DC14 (and Lys71 and Lys77 with DT10 and DC16), respectively (Fig. 2A and Movie S1). The major dissociation of the Sox2 protein from the *Fgf4* promoter occurred at 162 ps, and the N terminus dissociated completely at 300 ps by cutting off the interactions of Ser34, Lys35, and Asn8 with DT4, DT5, and DT6, respectively (Fig. 2A and Movie S2). The interaction of the C‐terminal loop was terminated along with the interaction of Arg73 and Arg75 with DG11 and DC16 and of Arg76 with DA15 bps (Fig. 2A and Table 1). In case of *Dppa4*, the dissociation started at the α2 interface at 180 ps and then proceeded to the α1‐helix region where the interactions of Ser34 and Lys35 with DT4; Arg15 with DA19; and Arg19 with DT20 were broken. The N‐terminal dissociation occurred at 320 ps, thereby restricting the interaction of amino acids with DT8 (Fig. 2A and Movie S3).

The mutant complexes also followed a pathway similar to that of the experimentally validated complexes 16. The mutant complex, F‐Mut‐TT, was experiencing fluctuations when the protein was pulled in the Y direction, and hence, it was necessary to pull it from the Z direction (the complex was rotated inside the simulation box, which made the orientation of Sox2 with its DNA distinct from the others). The major dissociation of this complex occurred at 200 ps, which was a considerably long time and the pulling direction was different from the rest of the complexes (Fig. 2B and Movie S4). Although the major dissociation took a long time, the C‐terminal loop was not able to dissociate completely from the DNA throughout the simulation. The mutated complex, F‐Mut‐GA, underwent major dissociation at 190 ps, while the C‐terminal residues (Thr80 and Lys77) were still maintaining interactions with the DNA backbone (DT1 and DT2). The major dissociation was mediated by breaking of the interactions of Asn8, Ser34, and Arg5 with DT6, DT5, and DC16 respectively. Moreover, in F‐Mut‐GA, there was no complete dissociation of Sox2 from DNA because the C terminus was in an uninterrupted interaction throughout the simulation (Fig. 2B and Movie S5). The C‐Mut‐AC complex started to dissociate at 150 ps and took a long time for its complete N‐terminal dissociation, while the C‐terminal loop started to dissociate after 700 ps by breaking the interactions of Tyr72 and Lys77 with DT16 and DA17, respectively (Fig. 2B and Movie S6).

**Figure 3:**
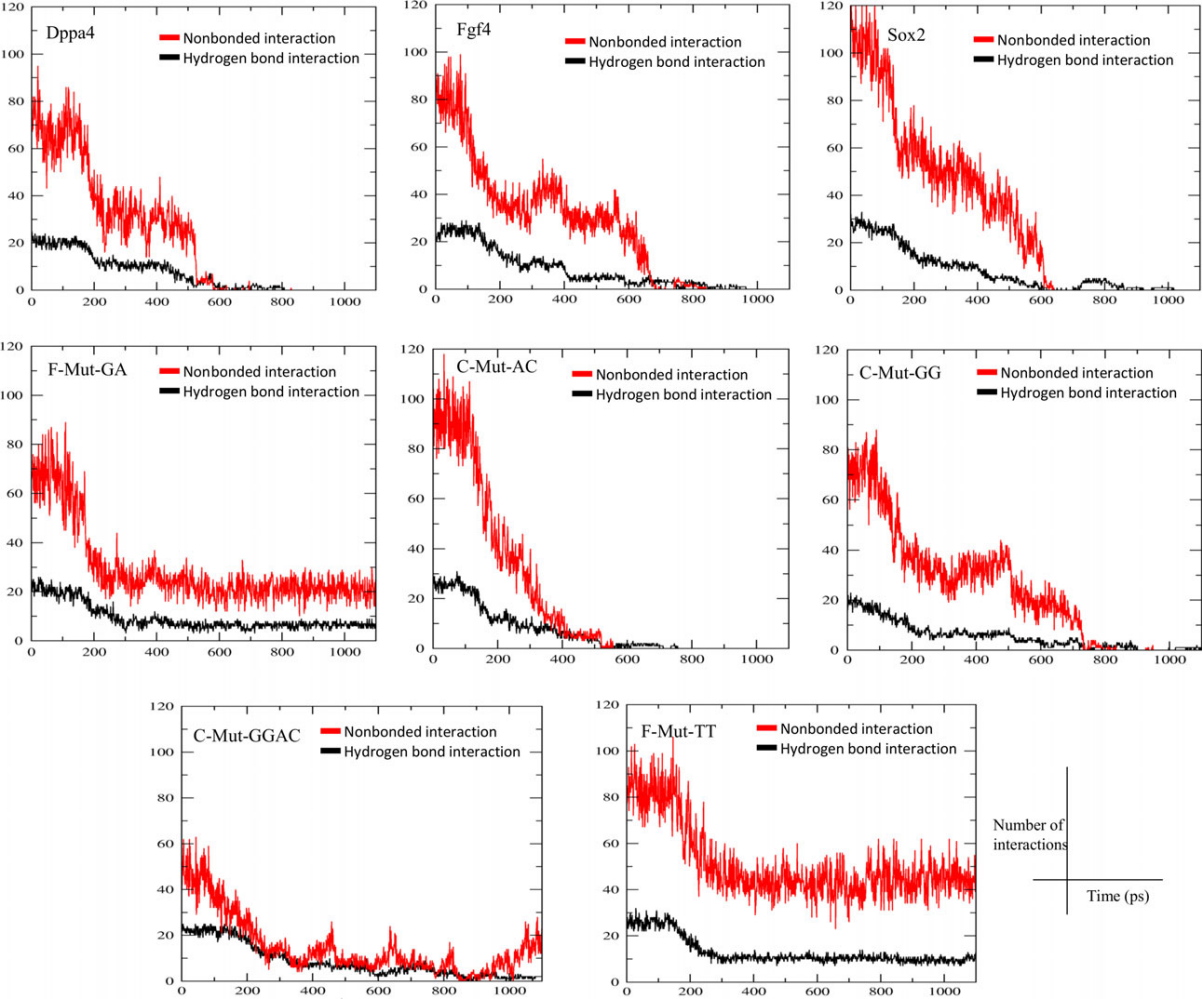
Hydrogen‐bonded and nonbonded interactions between the protein and DNA during the dissociation. The changes in the number of bonded (hydrogen bond) and nonbonded interactions between the Sox2 and DNA for all the complexes during the process of dissociation. Black and red represent the numbers of hydrogen‐bonded and nonbonded interactions, respectively.

C‐Mut‐GG and C‐Mut‐GGAC had their major dissociation events at 150 and 180 ps, respectively, while the C‐terminal Thr80 was in a continuous interaction with the backbone at DT1 and DT2 (Movies S7 and S8). The major dissociation observed for the C‐Mut‐GG complex was caused by termination of the interactions of Arg5 and Lys42 with DT8 and DG6, respectively, while for C‐Mut‐GGAC, the dissociation was observed during disruption of the interactions of Trp41 with DG6 and Arg5 with DC8 (Table 1). Additionally, the numbers of nonbonded interactions between the Sox2 protein and DNA during the dissociation process are calculated to characterize the binding affinity of the complexes and their behavior during the process (Fig. 3). The force and time of dissociation and a common representation of the dissociation pathway of the Sox2 protein can be seen in Fig. 2, and the interacting residues for all the complexes along with the time point of major dissociation and the force applied are summarized in Table 1.

During the process of dissociation, DNA was subjected to position restraint, and hence, the expected DNA relaxation from its bended state could not be observed. However, to verify the relaxation of DNA and to validate the process of dissociation, the dissociated Sox2 of one of the complexes, F‐Mut‐GA, was subjected to classic molecular dynamics (MD) simulation without position restraints. A snapshot obtained at 500 ps with the protein–DNA distance of 4.5 nm was simulated for 200 nanosecond (ns). It was observed that the DNA regained its relaxed state at the end of simulation, and the dissociated Sox2 tended to move toward the DNA for interaction. The centroid distance obtained between initial and final position of Sox2 at 200 ns was reduced to 2.54 nm (Fig. S3). Additionally, our previous study explained the relaxation of DNA after the removal of Sox2 20.

### The PMF profile

**Figure 4:**
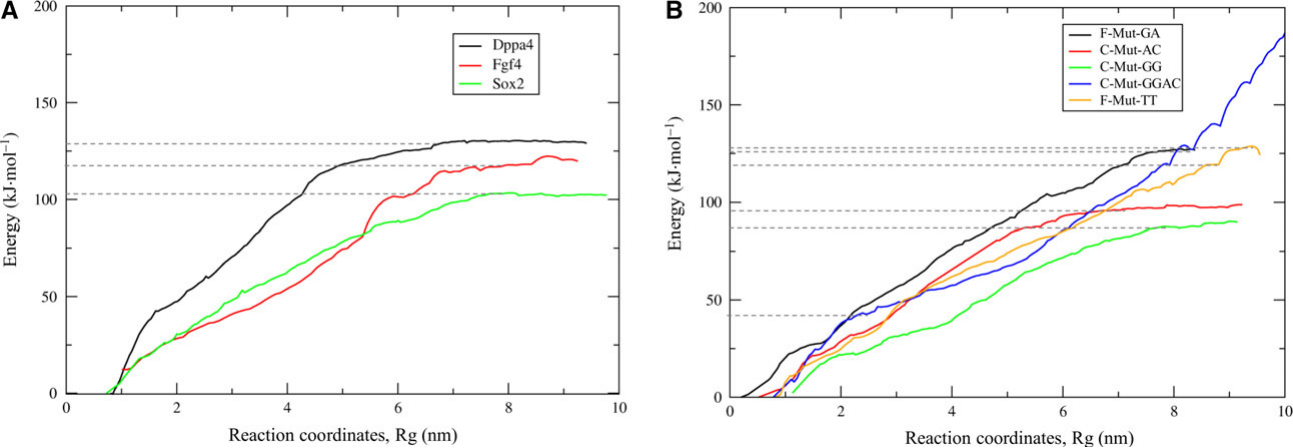
Binding free energy profiles obtained from the PMF calculation. (A) A comparison of binding free energies of experimentally validated complexes. (B) A comparison of binding free energies of mutant complexes.

The total interaction energy change (enthalpy) and entropy change throughout the reaction coordinates, Rg, of the Sox2 complexes are given by the PMF profile 21. This profile can be subdivided into two stages. At the first stage, the energy value was constantly increasing and represented the major dissociation of the Sox2 protein from DNA. The second stage has a flat profile corresponding to the process of C‐terminal loop dissociation of Sox2 (Fig. 4).

The PMF graph for *Dppa4* showed that the free energy gradually increased and reached a maximum value of 126.815 kcal·mol^−1^ at Rg of 6.6 nm. It was found that 98.6% of the total free energy changes for disassembly were spent on major dissociation, whereas the complete dissociation (second stage) occurred with the free energy of 128.569 kcal·mol^−1^ at Rg from 6.6 to 9 nm. In case of the *Fgf4* complex, the free energy spent to attain the local maximum point of major dissociation was 113.65 kcal·mol^−1^ at Rg of 6.9 nm, with almost 93.91% of free energy expended to reach this state. The second‐stage dissociation of the C terminus in this complex showed a constant flattened PMF up to 121.008 kcal·mol^−1^ until Rg of 8.8 nm. For the *Sox2* complex, there was a steady increase in the PMF value up to 103.151 kcal·mol^−1^ at Rg 8.1 nm, with the second‐stage PMF yielding a more flattened graph up to 103.58 kcal·mol^−1^ after 8.1 nm (Fig. 4). Although the *Sox2* complex releases its maximal energy at the greatest distance of separation during the dissociation, the PMF energy values are less than those of the other two complexes.

The PMF graph for F‐Mut‐GA showed a gradual increase in energy up to 124.78 kcal·mol^−1^ at Rg of 7.2 nm, which corresponded to 98.75% of its contribution, and the second stage of dissociation contributed ~ 126.354 kcal·mol^−1^. Meanwhile, the complex experienced another local minimum of 26.5 kcal·mol^−1^ at 1.8 nm. For the complex with F‐Mut‐TT, the local maximum point was reached at 123.7 kcal·mol^−1^ for its major dissociation (96.6%) at Rg of 8.8 nm. The second stage of PMF yielded an irregular graph with 127.93 kcal·mol^−1^ that contributed to 3.4% of the total free energy. C‐Mut‐AC showed free energy of 95.518 kcal·mol^−1^ at Rg of 6.7 nm (97.67% of total free energy), explaining that Sox2 required less binding energy for its association, and the second‐stage dissociation contributed 97.79 kcal·mol^−1^ (3.33% of total free energy).

The binding free energy for C‐Mut‐GG was 87.46 kcal·mol^−1^ at Rg of 7.8 nm, and the second‐stage contribution to its dissociation was 89.91 kcal·mol^−1^. The C‐Mut‐GGAC complex showed an agitated graph throughout the simulation, and the PMF energy value was 46.008 kcal·mol^−1^ at Rg of 2.9 nm center of mass (COM) distance during their first stage of dissociation. The major contribution of the energy was spent on the detachment of α2‐ and α1‐helices from the DNA for all the complexes, and the C‐terminal loop was being detached at the last stage. The free energy of dissociation for the Rg for all the complexes is depicted in Fig. 4.

The PMF for C‐Mut‐GGAC alone yielded an agitated graph that might be due to its secondary structure changes observed during the dissociation (Movie S8). To study the reversal of the secondary structure changes, the final snapshot of the dissociated Sox2 structure was simulated for 450 ns to verify the obtained results. During this time, the protein tried to refold into its original shape (Fig. S4). Although the 450 ns of simulation time is not sufficient to explain the whole refolding process, the comparison of the RMSD of the dissociated Sox2 structure and the simulated crystal structure may confirm that the protein tended to refold into its native conformation (Fig. S4). Further, to verify our reaction conditions, SMD was conducted at a lower force and rate, but this approach did not yield any different results. Another reason for the secondary structure changes may be the spring constant applied during SMD [1800 kcal·(mol·nm^2^)^−1^]. Hence, the force constant value was reduced to 50 kcal·(mol·nm^2^)^−1^, the pull rate was reduced to 0.005 nm·ps^−1^, and similar structural changes were observed for the complex (Movie S9).

### The impact of water molecules on dissociation of Sox2 from DNA

**Figure 5:**
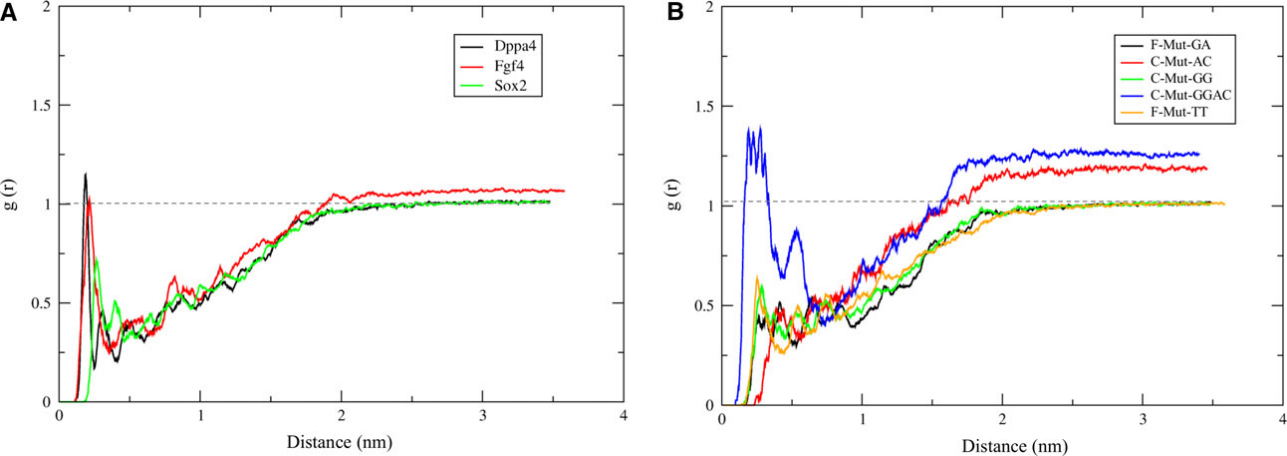
Radial distribution functions of water molecules around DNA. (A) RDF values of the experimental complexes showing hydration shells. (B) RDF values of mutant complexes showing hydration shells.

The radial distribution function (RDF) describes how atoms in a system are radially packed around each other, and an effective way to describe the disordered molecular systems. The behavior of the pulled Sox2 may affect the solvent molecules around DNA. The first solvation shell around DNA was located at ~ 0.1 nm for *Dppa4* and *Fgf4*, and at ~ 0.2 nm for *Sox2*, on the basis of the distances from water molecules. The sharp peak of RDF showed that the distributions of water molecules were highly ordered around the DNA. The second hydration shell appeared at ~ 0.3 nm for *Dppa4*, at ~ 0.42 nm for *Fgf4*, and 0.5–0.65 nm for the *Sox2* complex. There were no clear subsequent solvation shells after the second hydration shell. Around the distance of ~ 2.0–3.5 nm, the RDF reached the bulk value of ~ 1.0 (Fig. 5).

In case of mutant complexes, the first hydration shell was located at ~ 0.1 nm for F‐Mut‐GA, F‐Mut‐TT, and C‐Mut‐GG; at ~ 0.2 nm for C‐Mut‐AC; and at ~ 0.06 nm for C‐Mut‐GGAC. The RDF values of mutant complexes were found to be lower than the experimentally validated complexes 16. All the mutant complexes experienced a well‐pronounced peak for their first hydration shell except for C‐Mut‐GGAC. F‐Mut‐GA and F‐Mut‐TT complexes were found to have the second hydration shell at 0.4 and 0.6 nm, respectively. There were no clear subsequent hydration shells after the second hydration shell. The C‐Mut‐GGAC complex had the highest RDF value with the discarded peak in its first hydration shell, representing the chance that the orientation of water molecules around the DNA was not optimized. On the other hand, at the distance above ~ 1.8 nm (after major dissociation), the RDF reached its stable value of ~ 1.0 and gained its stable water molecule distribution (Fig. 5).

The water molecules serve as bridge between the protein and DNA, and it appeared that a delicate balance must be maintained to preserve the channel of water molecules that stabilize structure of the protein–DNA complex. Nevertheless, the disruption of native packing between protein and DNA may allow additional water molecules to interact with the amino acid residues. After reaching the maximal force, the interaction interface becomes entirely exposed to the bulk solvent (Fig. S5).

We needed to calculate the DNA sequence specificity for protein binding, it would be necessary to calculate the interactions mediated by the nitrogenous bases excluding the DNA backbone (sugar and phosphate groups). It was found that the whole system of all the complexes was trying to maintain its stable structure by incorporating more water molecules after the Sox2 removal (Fig. S5). Except for *Dppa4*, all experimental complexes showed a uniform increase in the number of water interactions with bps. The fifth and sixth (TT) bps of *Dppa4* were found to undergo a drastic change in the number of water molecule interactions. Similarly, for all the mutant complexes, the fourth and fifth bps showed a drastic increase in the number of water molecule interactions. Additionally, the F‐Mut‐TT and C‐Mut‐GG complexes yielded an abrupt increase in the number of water molecule interactions for their eighth and sixth bp positions, respectively. The highly conserved positions of all the complexes were affected by the Sox2 removal and hence showed the maximum of water molecule interactions. Because the RDF values of experimental complexes were higher, they required a smaller number of water molecules to optimize the structure when compared with the mutant complexes (Fig. 5 and Fig. S5).

### Base stacking energies

**Figure 6:**
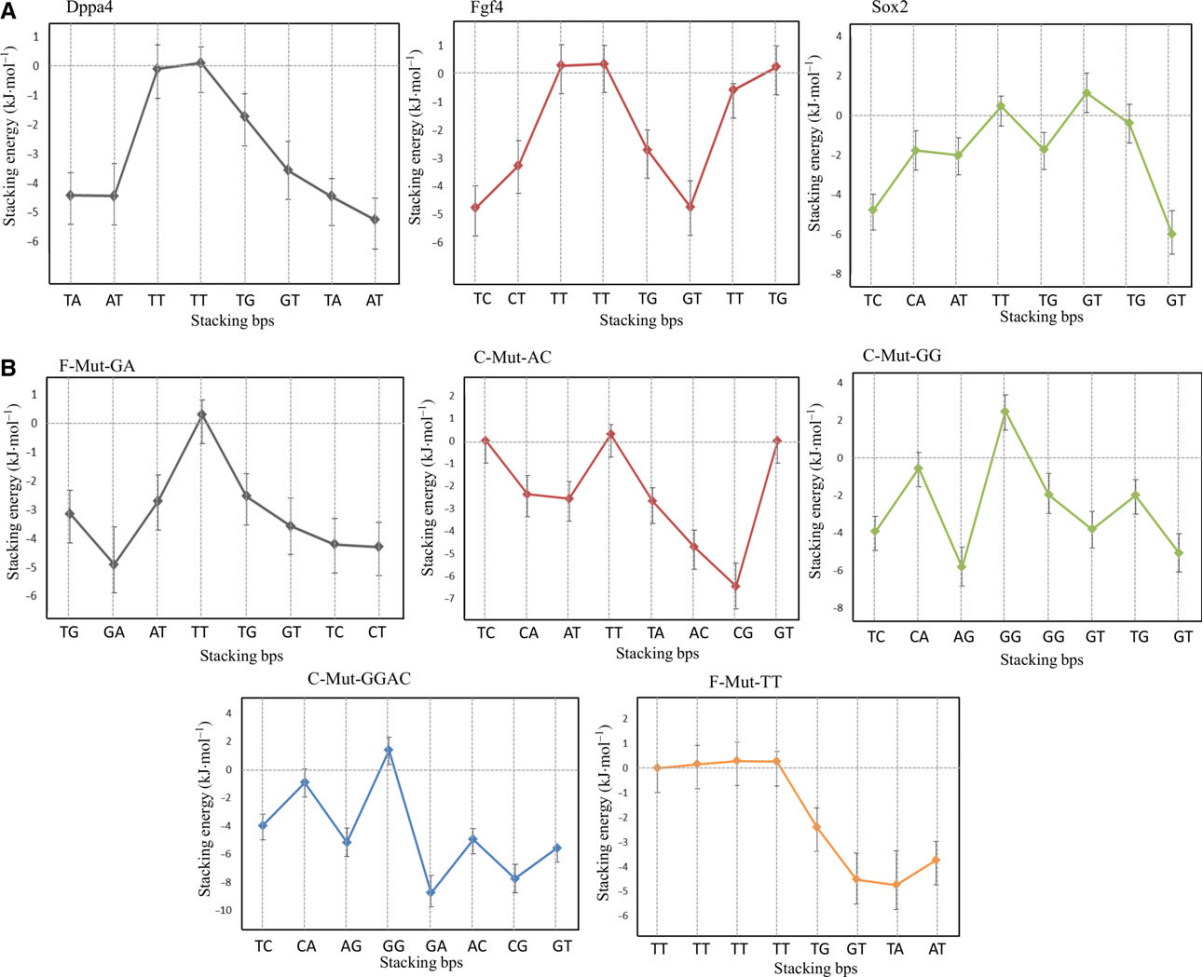
Stacking energies for DNA and its stability. (A) Stacking energies for experimentally validated DNA patterns showing higher values at fourth and fifth stacking bps. (B) Stacking energies for mutated DNA patterns showing increased stacking energies at fourth and fifth stacking bps in comparison with the positive control patterns. The stacking energies with its standard deviations are plotted in this figure.

The stacking interactions between bps are significant and contribute to the stability of the double helix. Stacking energy is a noncovalent force that stabilizes the stacking orientation and may play a greater role in DNA structure stability. The solvation effects that are likely to affect the stacking energetics depend on whether a DNA base (flat π‐surface) was better solvated by water or by an adjacent base's π‐surface 22. In general, the purines stack more strongly than pyrimidines because of its bigger surface area and polarizability. The stacking energies were calculated by means of the Motif Identifier for Nucleic acids Trajectory (mint) software, and the average values for each bp step are plotted in Fig. 6.

During the SMD simulation, DNA was subjected to position restraint (not all‐atom restraint); thus, we observed some atom oscillation and stacking energy changes during the simulation. *Dppa4* and *Sox2* enhancer complexes experienced lower base stacking energies in the flanking region. The third/fourth and fourth/fifth stacking bps were making foremost interactions with the dissociated Sox2 protein and hence showed fluctuation during the dissociation process with higher base stacking energies. The bp stacking energies of third/fourth and fourth/fifth stacking bps of these complexes ranged from −4.9 to +0.25 kcal·mol^−1^. In *Sox2* enhancer complex, the fourth/fifth and sixth/seventh stacking bps showed a higher stacking energy (1.2 kcal·mol^−1^). *Fgf4* showed higher stacking energy at eighth/ninth stacking bps, whereas the flanking regions of other complexes maintained lower stacking energy values. We know that if DNA undergoes any conformational change (bending or nicking), the stacking energies from the flanking region will try to optimize its stability 23, 24. Likewise, when the experimental complexes were under stress at third, fourth, and fifth position bps during the dissociation, the system could be optimized by the flanking region bp stacking energies.

In the mutant complexes, the flanking bp maintained lower stacking energy that supports its stability. C‐Mut‐GG and C‐Mut‐GGAC complexes were found to have a base stacking energy of > 2 kcal·mol^−1^ for their fourth/fifth stacking bps. F‐Mut‐GA and C‐Mut‐AC complexes showed stacking energies of ~ 0.15 kcal·mol^−1^ at the fourth/fifth stacking bps. As the pulling direction was different for the F‐Mut‐TT complex, the stacking energy profile observed was different from that of the other complexes. The first part of the profile showed energies from 0 to 0.26 kcal·mol^−1^, whereas the second part yielded lower stacking energies: from 0 to −4.89 kcal·mol^−1^. Hence, we found that the bp stacking energies for all the complexes were higher for fourth and fifth stacking bps (Fig. 6).

### Validation of binding affinities by the EMSA

We set up qualitative EMSA to estimate the binding affinities of these complexes. Three experimentally validated complexes (*Sox2*,* Dppa4*, and *Fgf4*) served as positive controls 16. Binding affinity values of conserved and flanking region mutant complexes were compared with the affinity of these positive controls. The flanking region with mutated GA bps (F‐Mut‐GA: GATTGTC) showed higher binding affinity as compared to the positive controls. Likewise, the F‐Mut‐TT (TTTTGTA) showed binding affinity similar to that of *Dppa4*. Moreover, C‐Mut‐AC (CATTACG) showed nonsignificantly higher binding affinity (Fig. S7). The complex with C‐Mut‐GG (CAGGGTG) showed a lower binding energy than did the positive controls. The negative control of this study, the complex with the mutant of the complete conserved region, C‐Mut‐GGAC (CAGGACG), showed the lowest binding energy in all the experiments, with an error value of 0.016. The validation of binding affinity data elucidated the greater importance of the flanking region similar to that of the conserved region in Sox2 binding.

### Pattern matching in the genome

One of the objectives of our study was to identify candidate target genes potentially regulated by Sox2. The mammalian genomes consist of noncoding sequence whose function is not well studied despite extensive research 25, 26, 27. The main portion of the noncoding sequence consists of enhancers that recruit sequence‐specific TFs (like Sox2), which in turn activate chromatin state, recruit coactivators, and therefore regulate the expression of the genes 28.

Our computational approach to the identification of the best binding patterns for Sox2 was validated via the pattern searching in the regulatory portion of the genome. The mutated and experimental patterns under study were used in searches against the limited human and mouse genome. Because the computational method was used to search for the patterns in the genome, it required stable input data. Hence, the list of target genes of Oct4, Sox2, and Nanog in human ESCs mentioned by Boyer *et al*. 17 served as a reference in this analysis. The genes were regarded as input for searches in the National Center for Biotechnology Information database to obtain gene IDs and sequence information. The experimental and the mutant Sox2‐binding patterns were searched against the full‐length sequence of each gene. As already reported, the Sox2‐binding site is located between positions −50 kb and +10 kb relative to the transcription start site (TSS) 28. The pattern searching was made using the fuzznuc software; the number of hits and locations of each matching pattern in the gene sequence was recorded. To narrow down the search, we also tried searching only for genes with an Oct4‐binding site adjacent to our Sox2‐binding patterns. Python scripts were utilized to screen these patterns, and these scripts are available in the figshare 29.

The pattern for *Dppa4* (ATTTGTA) was matched with 51 genes, among which the pattern occurred twice at different positions in five genes. The pattern for F‐Mut‐TT (TTTTGTA) was found in 121 genes, among which the pattern occurred twice at different positions in seven genes. The pattern matching F‐Mut‐GA (GATTGTC) was detected in 19 genes (Table 2). The fragile histidine triad (*FHIT*) gene matched both F‐Mut‐TT and *Dppa4* with two hits each. More than one hit for a pattern in a single gene raised the question which site binds first. This is an unsolved mystery for now; from our data, we can hypothesize that the priority of TF binding depends on the affinity values. The number of genes matching the binding patterns along with the number of hits for human and mouse genomes is presented in Table 2. The lists of genes whose patterns have been identified and sorted by the fuzznuc software are shown in Files S1 and S2.

**Table 2:** Sox2‐binding patterns in the human and mouse genomes identified in searches based on our DNA patterns

Complex	Pattern	Human		Mouse	
		Total number of genes matched	Genes with hit count greater than 1	Total number of genes matched	Genes with hit count greater than 1
*Dppa4*	ATTTGTA	36	*C20orf30, FHIT, MRPL47, NDUFB5*	15	*Efl1*
*Fgf4*	CTTTGTG	36	*KIF15*	16	–
*Sox2*	CATTGTT	36	–	6	–
F‐Mut‐GA	GATTGTC	12	–	7	–
C‐Mut‐AC	CATTACG	5	–	–	–
C‐Mut‐GG	CAGGGTG	36	–	9	–
C‐Mut‐GGAC	CAGGAC	2	–	2	–
F‐Mut‐TT	TTTTGTA	108	*DMD, DTNA, FHIT, GRID2, MAPRE2, NKX2‐3, SLC9A9*	13	–

## Discussion

Sequence‐dependent DNA structure and its flexibility as an alternative mechanism for recognition by proteins started gaining ground in recent years 30. Many TFs have the ability to recognize two distinct binding sites (primary and secondary) because of interdependence between the neighboring bps within a binding site 31. Sequence preferences of TFs are the prime mechanism by which the cell interprets the genome. Despite the central importance of these proteins in physiology, development, and evolution, DNA‐binding specificity has been characterized comprehensively in experiments for only a few proteins 32. This complexity of DNA recognition is significant in gene regulation and evolution of transcriptional regulatory networks.

The SMD results show a clear‐cut picture of the dissociation pathway for the Sox2 protein in all the complexes under our study. The overall dissociation mechanism consists of two stages: The first stage shows major dissociation of Sox2 from the DNA, yielding a rapid increase in the PMF value. The second stage of dissociation shows disconcerted PMF, which explains the dissociation of the C‐terminal loop of Sox2 from DNA (Fig. 4). It is known that amino acids such as Asp and Glu have greater affinity for adenine, whereas Pro and Phe residues are well known for the van der Walls interaction with thymine and adenine 33. Because the Sox2 protein does not contain many Asp, Pro, Glu, and Phe residues in the α1 and α2 regions, the major dissociation of Sox2 occurs more quickly, thereby causing a rapid increase in PMF energy during the first stage for most of the complexes under study. Nevertheless, the C‐terminal loop of Sox2 is rich in Arg and Lys residues and is wrapped around DNA. Arg and Lys are widely distributed around DNA bases likely because the long side chains of these amino acids can accommodate different positions and form hydrogen bonds with a base 11, thereby making the C‐terminal dissociation process complicated and yielding the flat sluggish PMF graph for the second stage of dissociation. The PMF graph for the C‐Mut‐GGAC complex shows a distorted curve (Fig. 4, blue curve) due to the major secondary structure changes (unfolding) observed in Sox2 during the dissociation. A similar result in the PMF graph has been observed in the unfolding process of proteins 34. When a mutation occurs in the highly conserved region of a Sox2‐binding site (Mut‐GGAC), the complex loses its affinity, and its structure is disrupted. In addition, it was confirmed here that the distorted curve is due to the secondary structure changes, not the pulling rate and force (Movie S9). Moreover, the refolding process of the disrupted Sox2 structure was monitored for 450 ns, and the structural changes are presented in Fig. S4.

During umbrella sampling, the maximal force at which the dissociation occurs and time required to achieve the maximal force depend upon the pulling rate because the structural conformations occur at different rates 35. Therefore, the time and force required by each complex for its dissociation are distinct for different complexes (Fig. 2 and Table 1). The dissociation time for F‐Mut‐GA and F‐Mut‐TT is longer when compared with the other complexes, and the force constant required for their dissociation is the same as that for the experimental complexes (*Dppa4* and *Fgf4*). The force constant and dissociation time are proportional to the bonding interaction between the protein (Sox2) and DNA in the complexes (Figs 2 and 3, Table 1). This bonding intensity corresponds to the binding preference for the Sox2. Sox2 also requires high energy (PMF) to dissociate from its preferred binding pattern, and it can be translated to the longer time requirement to dissociate.

A single incorrect or mutant bp is expected to reduce the favorable free energy of specific binding by 2–3 kcal·mol^−1^ 36. Even though the dissociation pathways of *Dppa4*,* Fgf4*,* Sox2*, and the mutant complexes are similar (differ by one or two bps in conserved and flanking regions), the binding free energies are different. The PMF graph for *Dppa4*,* Fgf4*, and *Sox2* shows that the maximal free energies of dissociation are 126.815, 113.65, and 103.151 kcal·mol^−1^ respectively, and reach a local maximum point at Rg of 6.6, 6.9, and 8.1 nm, respectively. In the case of mutant complexes, F‐Mut‐GA and F‐Mut‐TT show a similar but higher binding free energy of 124.78 and 123.7 kcal·mol^−1^ at Rg of 7.2 and 8.8 nm, respectively. The maximum interaction energy (PMF) spent, the maximum dissociation time, and the maximum force applied for the dissociation of Sox2 from DNA have been observed for these complexes. This suggests that the Sox2 will have higher binding preference for these mutant complexes that have also been observed in experimental complexes. Although these two mutant complexes spend more energy on the major dissociation, the C terminus of Sox2 does not dissociate completely from the DNA throughout the simulation (Movies S5 and S4, respectively). The C‐terminal region interacts with the DNA continuously, and the nonbonded interactions are maintained between the protein and DNA until the end of simulation, as shown in Fig. 3, thereby proving that these complexes are more stable. Thr80, Arg76, and Lys77 are the major residues forming interactions with the DNA backbone at DT1, DT2, and DT2 positions, respectively. Purines have a strong preference for few amino acids (Arg, Lys, Gln, and Asn), whereas pyrimidines show large variations in their preferences. Furthermore, the scoring matrix of Mandel *et al*. 8 provides log odd values for each amino acid with its DNA bp interactions. Based on these reports, highly purine–pyrimidine‐rich bp patterns (F‐Mut‐GA, F‐Mut‐TT, and *Dppa4*) may capable of maintaining the interactions with Sox2 better than other bps can during dissociation. Therefore, the binding pattern preference of Sox2 can be greater for F‐Mut‐GA and lesser for C‐Mut‐GG complexes (F‐MutGA > F‐Mut‐TT > Dppa4 > Fgf4 > C‐Mut‐AC > Sox2 > C‐Mut‐GG > C‐Mut‐GGAC). Moreover, Sox2–DNA binding affinity values are assessed by an EMSA, which proves that the flanking region mutated complexes (F‐Mut‐GA and F‐Mut‐TT) expresses higher binding affinity values than the experimentally validated complexes 16 (Fig. S7).

Proteins use electrostatic (including hydrogen bonds) and van der Waals interactions to overcome the energy penalty for distortion, with the optimal sequences requiring less deformation energy. Protein–DNA interaction energy (especially van der Waals) correlates well with the loss of the solvent‐accessible surface area of DNA upon protein binding 30. Therefore, all the complexes showed an increased solvent‐accessible area for DNA after the removal of Sox2 during the dissociation process (Fig. S6). Protein–DNA recognition is governed mainly by electrostatic interactions, implying that the aqueous environment plays an important role in protein–DNA interactions.

The RDF peaks represent the preferential orientation of water molecules around the DNA 37, and the dissociation of Sox2 has a major impact on the hydration shells around the DNA. Once the dissociation starts, the highly ordered water molecules will lose their orientation and will start to unpack. The intensity of peaks of the subsequent hydration shells is lower with a decrease in the distribution of water molecules and causes poor solvation of DNA until the distribution of water molecules regains its bulk value. The mutant and experimental complexes show similar behavior of the RDF function. All the complexes yielded a clear‐cut first hydration shell except for C‐Mut‐GGAC owing to the poor orientation of water molecules around the DNA. The distribution of water molecules reached its optimized orientation for F‐Mut‐GA and F‐Mut‐TT complexes, thereby trying to form a stable network for the corresponding DNAs (Fig. 5). The RDF values correlate with the number of water molecules required by DNA bps in order to obtain a stable structure during the dissociation process, and we found that the number of water molecules required for mutant complexes is higher than that for the experimental complexes (Fig. 5 and Fig. S5).

Even though a stable protein–DNA complex does not require an ample amount of water‐mediated interactions for its stability, it requires a huge number of water‐mediated interactions when there is a stress in the association. The fourth, fifth, and sixth intercalating stacking bps of all complexes show a larger number of water molecule interactions helping to retain stability during the dissociation process (Fig. S5). Water molecules residing in the minor groove of DNA facilitate the insertion of arginine side chains 38. Intercalation of amino acid side chains into the DNA helical stack promotes stabilization of the DNA substrate via protein–DNA interactions mimicking base–base stacking 39. The higher base stacking energy values at fourth, fifth, and sixth conserved stacking bps are due to the major dissociations taking place at these positions and intercalation of Arg residues. In the *Sox2* enhancer complex, the bp intercalation occurs between sixth and seventh stacking bps, thereby increasing stacking energy (Fig. 6 and Movies [Link], [Link], [Link], [Link], [Link], [Link], [Link], [Link], [Link]). In other studies, researchers found that the unequilibrated stacking energies of bps are optimized by stacking energies of the flanking region 24. Thus, for all the complexes other than the *Fgf4* enhancer complex, the flanking region stacking energies are maintained at a lower level, thereby supporting the stability. Because purines contain heavier atoms, they have larger Lennard‐Jones (L‐J) potential contributions and hence less stacking free energy 40. Accordingly, stacking free energies of nucleotides can be ranked as follows: purine–purine > purine–pyrimidine > pyrimidine–pyrimidine, with lower stacking free energy for higher stability. F‐Mut‐GA, F‐Mut‐TT, and *Dppa4* show lower stacking energies and hence contribute to the DNA stability (Fig. 6).

The overall analysis of the dissociation pathway, PMF value (binding affinity), base stacking energy, radial distribution, and the water‐mediated interactions for the distinct DNA patterns revealed that F‐Mut‐GA, F‐Mut‐TT, and *Dppa4* are more stable than the other patterns. Hence, it can be mentioned that patterns ATTTGTA (*Dppa4*), GATTGTC (F‐Mut‐GA), and TTTTGTA (F‐Mut‐TT) are better binding sites for Sox2. In addition, we can conclude that the flanking regions in a binding pattern for Sox2 are important just like the conserved region. The α2 region of a Sox2 interacting with the mutated flanking region requires more energy for its dissociation, indicating that the binding is stronger. The stronger binding of the α2‐helix validates the binding affinity of the complex. Even a single bp alteration in the flanking region affects the Sox2 binding affinity and may cause variation in its function.

It has been reported that the binding patterns obtained from databases and from experimental results are different 13. Furthermore, specific sequences to which the Sox2 protein binds *in vivo* only partially match the *in vitro* consensus 41. As explained in Results, in order to enhance the accuracy of binding pattern preference of Sox2, it is necessary to analyze and sort the target genes that match with its distinct binding patterns. The screening of the binding patterns for Sox2 in the genome yielded a sorted list of Sox2 target genes, locations, and the numbers of occurrences of a pattern. We found that the occurrence of patterns F‐Mut‐TT (TTTTGTA) and *Dppa4* (ATTTGTA) is highly frequent in ESC‐related genes (Table 2). The occurrence of more than one hit appears highly favorable for these patterns, suggesting that Sox2 binds to an alternative site and mediates different responses. For example, the *FHIT* gene shows two hits for both F‐Mut‐TT and *Dppa4* and plays a major role in differentiating humans from apes 42. To date, the unanswered question is how a TF selects a specific binding site between the two similar binding sites present in a single gene. Selecting an appropriate binding site is vital for regulation of the expression of genes 29. The selection criteria always depend on the cellular environment (binding partners and other components), protein–DNA dynamic conformational changes, and the tight packing of TF with its DNA pattern (affinity). The calculation of affinity values of distinct binding patterns may help to determine which pattern (binding site) will be prioritized by the TF. Similarly, Merino *et al*., performed MD simulation studies to estimate the relative cooperative binding free energies of Oct4 with Sox2 and Sox17 in the canonical and compressed composite motifs, respectively. The authors demonstrated that the MD simulation methods can be employed to study the cooperative DNA recognition 43.

Even though the whole genome contains more than a half of noncoding genes, our pattern searching can be a good screening for specific TFs, and identification of the target genes for the TFs will be useful. These computationally inexpensive methods should facilitate identification of unknown target genes for various other TFs and will help investigators to narrow down the search for transcriptional regulatory circuitry in ESCs. It would be interesting to study the occupancy of preferential binding sites of Sox2, Oct4, and Nanog in various genes. These data in turn will provide more control over the procedure inducing pluripotency in cells.

## Materials and methods

### Initial structure modeling and mutation

The HMG domain structure of Sox2 was obtained from Protein Data Bank (ID: 1GT0), and missing residues were modeled and minimized using chimera (Biocomputing, Visualization, and Informatics, University of California, San Francisco, CA, USA). Terminal charges were applied before the simulation. Based on the higher number of occurrences of patterns in common, three experimentally validated Sox2‐binding patterns (*Fgf4*,* Sox2*, and *Dppa4*) were selected among the genes mentioned by Yusuf *et al*. 16. The mutant DNA patterns were obtained by means of the ‘build and edit nucleic acid’ module in the Discovery Studio visualization package. Because AT and GC bps are almost identical in size and dimensions and occupy the same amount of space in a DNA double helix 44, transversion of a double‐stranded DNA binding site is an ideal object to obtain appropriate mutated binding patterns. The mutant protein–DNA models were obtained after the superimposition of the Sox2 crystal structure (1GT0), and the alignment was made with the reference to the backbone of double‐stranded DNA (both strands). Because not all the possible combinations of binding patterns could be analyzed in a single attempt, we considered five mutant complexes that included the alteration in highly conserved and flanking regions of a Sox2‐binding pattern. The selection of bp for mutation at the conserved and flanking regions of Sox2‐binding pattern was based on the lowest and the highest chances of its occurrences, respectively, as indicated in sequence logo obtained from the JASPAR database 45. Hence, eight binding patterns were analyzed to understand the variability in their affinities. *Dppa4* (ATTTGTA), *Fgf4* (CTTTGTT), and *Sox2* (CATTGTG) are experimentally proven DNA motifs for Sox2 binding, whereas F‐Mut‐GA (GATTGTC), C‐Mut‐AC (CATTACG), C‐Mut‐GG (CAGGGTG), C‐Mut‐GGAC (CAGGACG), and F‐Mut‐TT (TTTTGTA) are the complexes with mutated patterns (Fig. 1 and Fig. S1). The notation C‐Mut indicates a mutation in the central 4‐mer bases, while F‐Mut indicates a mutation in the flanking region bases. The structures of these complexes (protein–DNA) were minimized using Chimera by applying a conjugate gradient and the steepest descent methods.

### Molecular dynamics simulation

The reported and mutated complexes were subjected to MD simulation in order to remove the steric clashes obtained during the model building and to analyze the structure stabilities. All the complexes were minimized and subjected to MD simulation for 10 ns, and the RMSD curves are plotted in Fig. S2. The simulation was performed in gromacs 4.6 46 with AMBERff‐99SB‐ILDN force field 47 at 150 mm NaCl along with the TIP3P water model to solvate the cubic box (7 × 7 × 7); periodic boundary conditions were also applied 48. Although the later‐generation force fields (parmbsc1 and parmbsc0) that correct anomalous backbone transitions in long simulations have been improved, these force fields will not add any benefit to our short step steered MD simulations. The whole system was minimized with a maximal force tolerance of 1000 kcal·(mol·nm)^−1^. Two‐step equilibration was conducted for each complex, and the equilibrated systems were subjected to a production MD simulation. The last snapshot of a 10‐ns simulation was taken as an initial structure for the SMD simulation. The detailed protocol for MD simulation has been provided in our previous papers 49.

### Steered MD and umbrella sampling

The starting structures (10‐ns snapshots of all‐atom MD simulation) were placed in a rectangular box with the dimensions that were sufficient for the pulling simulations to take place along the Y direction (7 × 27 × 7 nm). The box was filled with TIP3P water molecules, and 150 mm NaCl was added to maintain the physiological ion conditions along with necessary counterions to neutralize the system. The total number of water molecules was ~ 31 000, and the total number of atoms in the whole system was ~ 35 000. The whole system was minimized and equilibrated with a force constant of 1000 kcal·(mol·nm)^−1^ of the steepest descent. A Nose‐Hoover thermostat 50, 51 maintained the temperature, and a Parrinello‐Rahman barostat 52 was used for maintaining pressure. The cutoff for the van der Waals interactions was 1.0 Å. Nucleic acids are highly charged molecules, can interact strongly with their solvent and other solutes over long distances. Long‐range electrostatic forces may greatly influence the delicate balance of structural forces in conformations of nucleic acids. Thus, the particle mesh Ewald method was used for proper treatment of long‐range electrostatic interactions. Under physiological pressure, the whole system was equilibrated for 100 ps. After the equilibration, the restrains were removed from the protein molecule to make it a pulling group, whereas DNA was regarded as an immobile reference group with the position restrains. The protein molecule was pulled away from the DNA for 1100 ps using the spring constant of 1800 kcal·(mol·nm^2^)^−1^ and a pull rate of 0.008 nm·ps^−1^ (8 nm per 1 ns). The SMD simulation was carried out for 1100 ps for each of the eight complexes under study.

From the trajectories, the snapshots with a window spacing of 0.2 nm up to the 8.8‐nm COM of protein and DNA separation were collected for umbrella sampling, resulting in ~ 35–45 windows for each complex. Each window underwent a 5‐ns simulation. Hence, each of the eight complexes underwent a simulation for 175–225 ns. The free energy profile along the separation coordinate was obtained from the combined population densities of the simulation windows, and instantaneous values of the biasing potential were obtained using the weighted histogram analysis method 53, 54.

### PMF calculation

Potential of mean force can be calculated from the system energy changes as a function of reaction coordinates. PMFs can be used to represent the energetics of a range of biological systems of interest, such as protein folding and unfolding, interactions between molecules, and conformational changes within a molecule 55. Reconstruction of the PMF from SMD simulations is based on well‐known Jarzynski's equality 56, 57, 58, which connects the nonequilibrium work values (*W*) in driving a system initially to equilibrium with the change in free energy between the initial and final state (Δ*F*) through the nonlinear average as shown below:

(1) $$\left\langle e^{-\beta W}\right\rangle =e^{-\beta \Delta F}$$

where $\beta ={\left(k_{B}T\right)}^{-1}$ denotes the inverse temperature and *k*_B_ is the Boltzmann constant; 〈〉 is over‐repeated realizations of the progress and represents an average across all possible realizations of an external process that takes the system from the equilibrium state to a nonequilibrium state under the same external conditions.

### DNA base stacking energy

The base stacking energy of all the complexes with distinct binding patterns was estimated by means of the mint software 59. Base pairing between complementary strands and stacking between adjacent bases are the main factors that determine DNA stability and shape 23. mint provides the sum of van der Waals and the electrostatic interactions per nucleotide as an average stacking energy. The stacked bp, that is, the adjacent nucleotides inside the double helix interact with each other via van der Waals forces and electrostatic interaction and contribute to the stability of the double helix. mint splits the input trajectory into pieces of equal time spans and analyzes each subtrajectory on a separate core at the same time. Finally, the software computes statistics for all frames and provides the output 59.

### emboss fuzznuc: identification of a Sox2‐binding site in gene enhancer regions

The mutated and experimental binding patterns of Sox2 analyzed in our study were searched for its existence in the whole mouse and human genomes. Boyer *et al*. identified the target genes for Oct4, Sox2, and Nanog from human ESCs by genome‐scale location analysis. The list of possible ESC‐related target genes of Sox2 was obtained from the above study 17, and their binding sequences were analyzed for the possibility of matching with our identified mutant patterns. The pattern matching was conducted by means of the emboss fuzznuc program 60, 61. fuzznuc searched for a specified short pattern in nucleotide sequences. Experimental reports suggest that the canonical and compressed motifs of Sox2 were located ~ 10–50 kb away from the TSS 28, and hence, the searching was made between positions –50 kb upstream and +10 kb downstream of a TSS in the genes. The total sequence of each gene was fed as an input using a python script 29. The program could search for an exact pattern or match variable lengths of patterns and repeated subsections of the sequence. The output was a standard EMBOSS report file that includes data such as location of the pattern, gene ID, total length of the gene searched, and the score of any matches/hit counts 60, 62.

## Author contributions

DY and SC planned experiments. DY and H‐KK performed experiments. DY, MAA, and SP analyzed data. SC contributed for material. DY, MAA, and SC wrote the manuscript.

## Supporting information


**Fig. S1.** Demonstration of the initial structure and mutated complexes for Sox2.
**Fig. S2.** Molecular dynamics simulation of the modeled complexes subjected to umbrella sampling.
**Fig. S3.** Validation of dissociation process and relaxation of DNA.
**Fig. S4.** Process of refolding of disrupted Sox2 in the complex with C‐Mut‐GGAC.
**Fig. S5.** Water‐mediated interaction with bps of DNA.
**Fig. S6.** Comparison of the solvent‐accessible surface areas (SASAs) during dissociation.
**Fig. S7.** EMSA experiment intended to determine binding affinity.
**Table S1.** A list of EMSA oligonucleotide sequences.

**File S1.** Sox2 binding patterns matching with human ESC target genes.

**File S2.** Sox2 binding patterns matching with mouse ESC target genes.

**Movie S1.** Dissociation of Sox2 from DNA in the *Sox2* promoter (CATTGTG).

**Movie S2.** Dissociation of Sox2 from DNA in the *Fgf4* promoter (CTTTGTT).

**Movie S3. Dis**sociation of Sox2 from DNA in the *Dppa4* promoter (ATTTGTA).

**Movie S4.** Dissociation of Sox2 from DNA in F‐Mut‐TT (TTTTGTA).

**Movie S5.** Dissociation of Sox2 from DNA in F‐Mut‐GA with (GATTGTC).

**Movie S6.** Dissociation of Sox2 from DNA in C‐Mut‐AC (CATTACG).

**Movie S7.** Dissociation of Sox2 from DNA in C‐Mut‐GG (CAGGGTG).

**Movie S8.** Dissociation of Sox2 from DNA in C‐Mut‐GGAC (CAGGACG).

**Movie S9.** Dissociation of Sox2 from C‐Mut‐GGAC (CAGGACG) with a force constant of 50 kcal·(mol·nm^2^)^−1^.
