# Supplementary material for: Evaluation of Sox2 binding affinities for distinct DNA patterns using steered molecular dynamics simulation
Source: FEBS Open Bio. 2017 Oct 9;7(11):1750–67. doi: 10.1002/2211-5463.12316 (PMC5666385; doi:10.1002/2211-5463.12316)
Supplement: Supplementary file 1 — Fig. S1. Demonstration of the initial structure and mutated complexes for Sox2. Fig. S2. Molecular dynamics simulation of the modeled complexes subjected to umbrella sampling. Fig. S3. Validation of dissociation process and relaxation of DNA. Fig. S4. Process of refolding of disrupted Sox2 in the complex with C‐Mut‐GGAC. Fig. S5. Water‐mediated interaction with bps of DNA. Fig. S6. Comparison of the solvent‐accessible surface areas (SASAs) during dissociation. Fig. S7. EMSA experiment intended to determine binding affinity. Table S1. A list of EMSA oligonucleotide sequences. [file FEB4-7-1750-s001.pdf]

<Supplementary Information>

Evaluation of Sox2 binding affinities for distinct DNA patterns by steered molecular dynamics simulation

Dhanusha Yesudhas, Muhammad Ayaz Anwar, Suresh Panneerselvam, Han-Kyul Kim & Sangdun Choi*

Department of Molecular Science and Technology, Ajou University, Suwon, 16499, Korea

***Correspondence**
Sangdun Choi
Department of Molecular Science and Technology, Ajou University, Suwon, 16499, Korea
Fax: +82 31-219-1615
Tel: +82 31-219-2600
E-mail: sangdunchoi@ajou.ac.kr

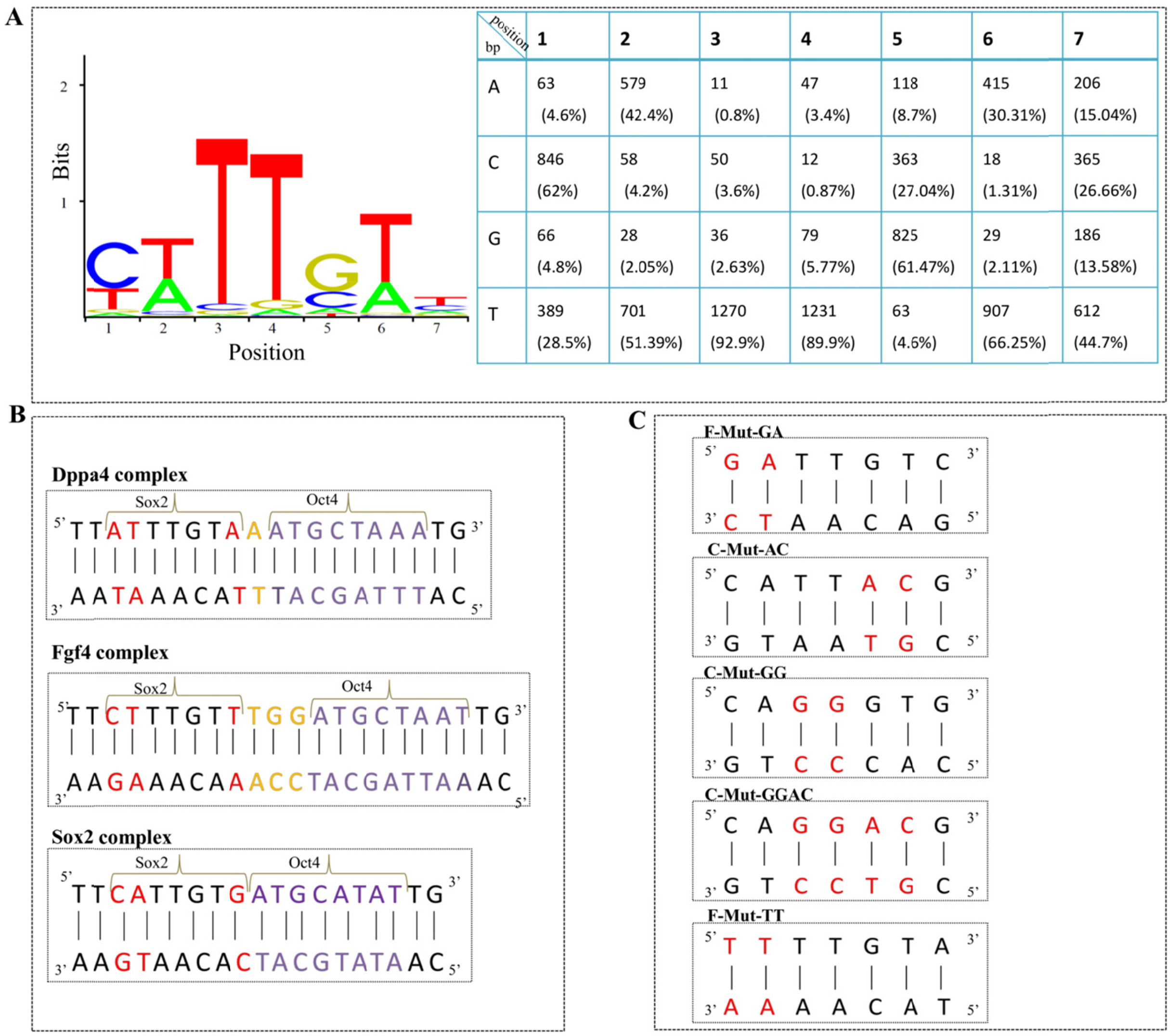

Position / bp	1	2	3	4	5	6	7
A	63 (4.6%)	579 (42.4%)	11 (0.8%)	47 (3.4%)	118 (8.7%)	415 (30.31%)	206 (15.04%)
C	846 (62%)	58 (4.2%)	50 (3.6%)	12 (0.87%)	363 (27.04%)	18 (1.31%)	365 (26.66%)
G	66 (4.8%)	28 (2.05%)	36 (2.63%)	79 (5.77%)	825 (61.47%)	29 (2.11%)	186 (13.58%)
T	389 (28.5%)	701 (51.39%)	1270 (92.9%)	1231 (89.9%)	63 (4.6%)	907 (66.25%)	612 (44.7%)

Fig. S1. Demonstration of the initial structure and mutated complexes for Sox2. (A) The sequence logo for a Sox2-binding site obtained from the JASPAR database along with the table showing the frequency (percentage) of occurrence of each bp at different positions. The figure was obtained from the JASPAR database and modified. (B) DNA patterns for experimentally proven Sox2- and Oct4-binding sites used in our analysis. The conserved and flanking regions are black and red respectively, for Sox2, and the Oct4-binding site is purple. The spacing between Oct4 and Sox2 in experimentally validated complexes is yellow. (C) Mutant DNA patterns of Sox2-binding sites used in our analysis. Mutations in bps are highlighted in red.

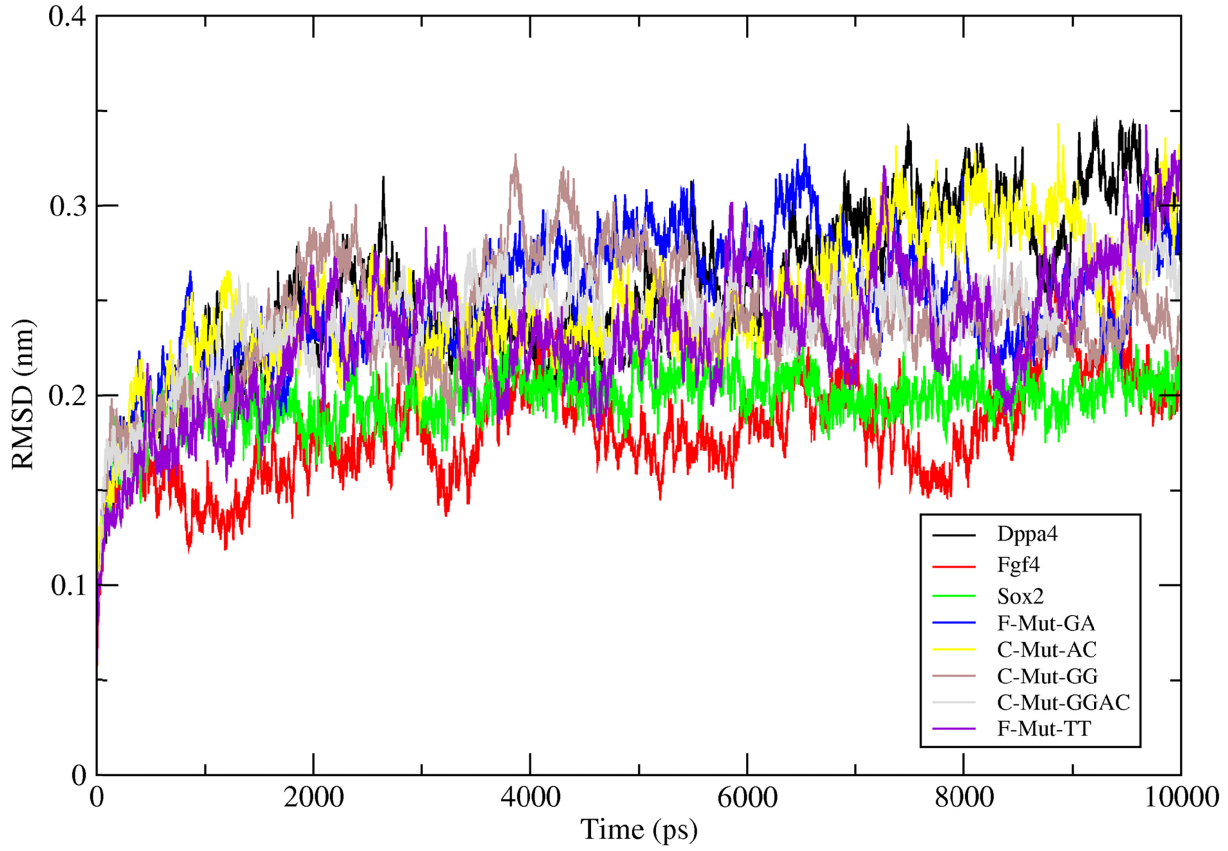

Fig. S2. Molecular dynamics simulation of the modeled complexes subjected to umbrella sampling. A root mean square deviation (RMSD) graph for the backbone atoms of the positive-control and mutant complexes used for umbrella sampling. The complexes were modeled using Discovery Studio, and minimized as well as simulated for 10 ns to obtain an optimized model for umbrella sampling.

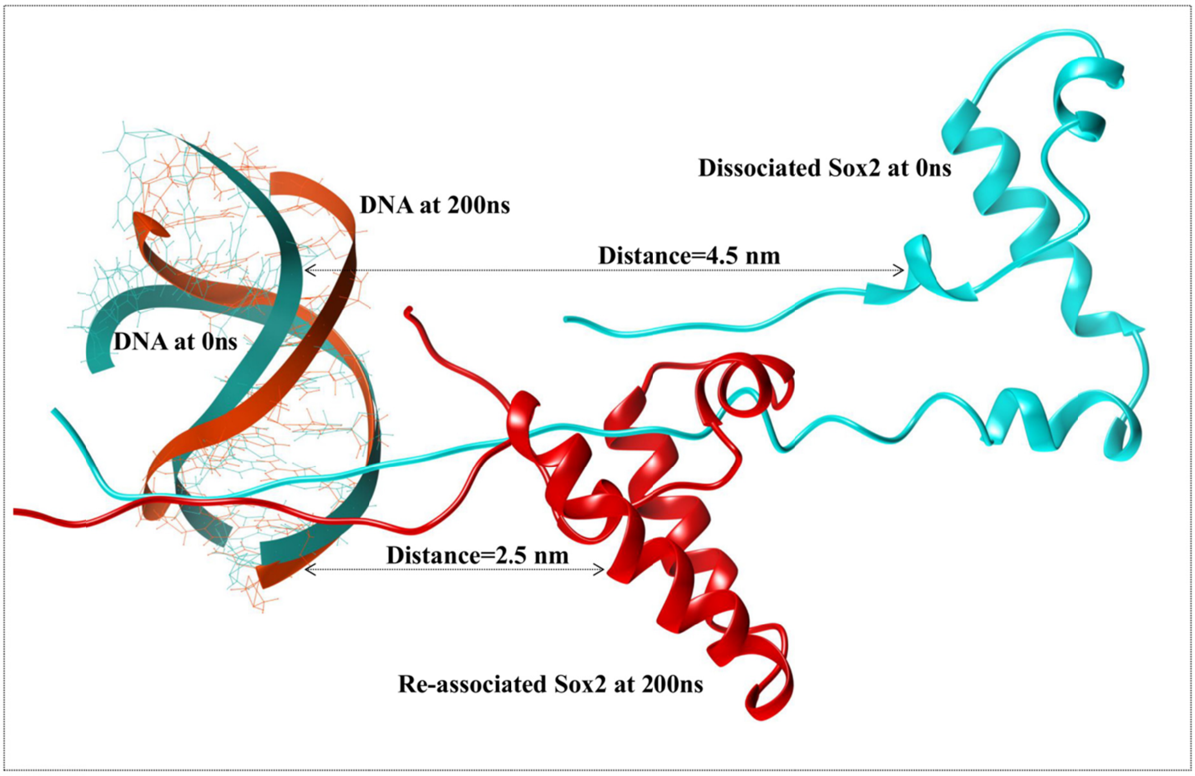

Fig. S3. Validation of dissociation process and relaxation of DNA. Simulation of the dissociated Sox2 for 200 ns without position restraints showing the relaxation of DNA, and the tendency of Sox2 moving towards the DNA for interactions. F-Mut-GA complex with Sox2-DNA at 0 ns and a distance of 4.5 nm is shown in cyan, and Sox2-DNA at 200 ns with the reduced distance of 2.5 nm is shown in red.

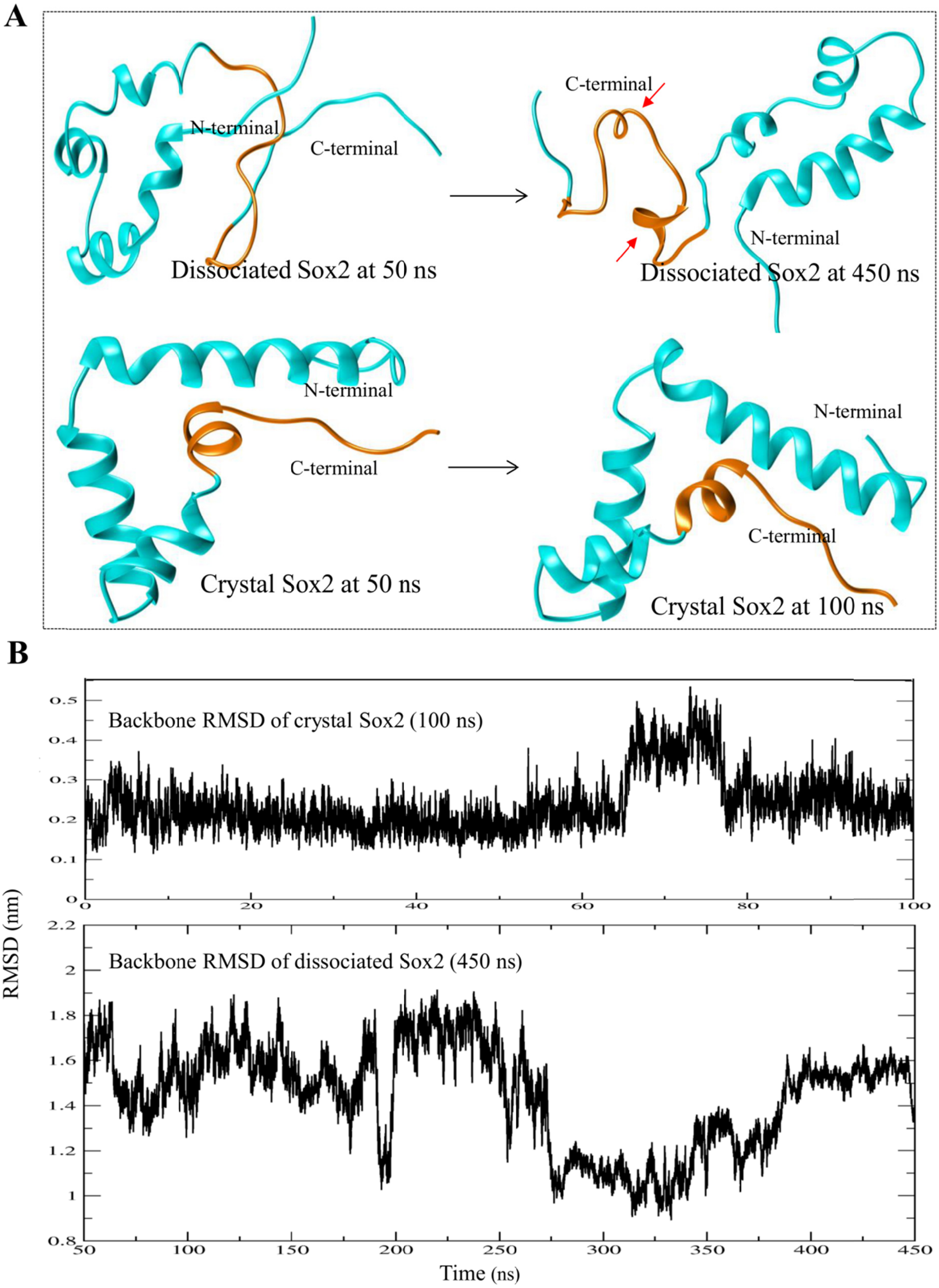
A
N-terminal
C-terminal
Dissociated Sox2 at 50 ns
C-terminal
N-terminal
Dissociated Sox2 at 450 ns
N-terminal
C-terminal
Crystal Sox2 at 50 ns
N-terminal
C-terminal
Crystal Sox2 at 100 ns
B
Backbone RMSD of crystal Sox2 (100 ns)
Backbone RMSD of dissociated Sox2 (450 ns)
RMSD (nm)
Time (ns)

Fig. S4. Process of refolding of disrupted Sox2 in the complex with C-Mut-GGAC. (A) Comparison between the processes of refolding of Sox2 dissociated from the C-Mut-GGAC complex that had undergone secondary structure changes and crystal Sox2 during the 50-ns and 100-ns simulations. The disrupted Sox2 structure tries to attain the original structure during the simulation. The red arrow indicates the highly disturbed α3 region, trying to regain its helical shape during the simulation. (B) Comparison of the RMSD graphs of crystal Sox2 (top) and Sox2 dissociated from the C-Mut-GGAC sequence (bottom) that had undergone secondary structure changes.

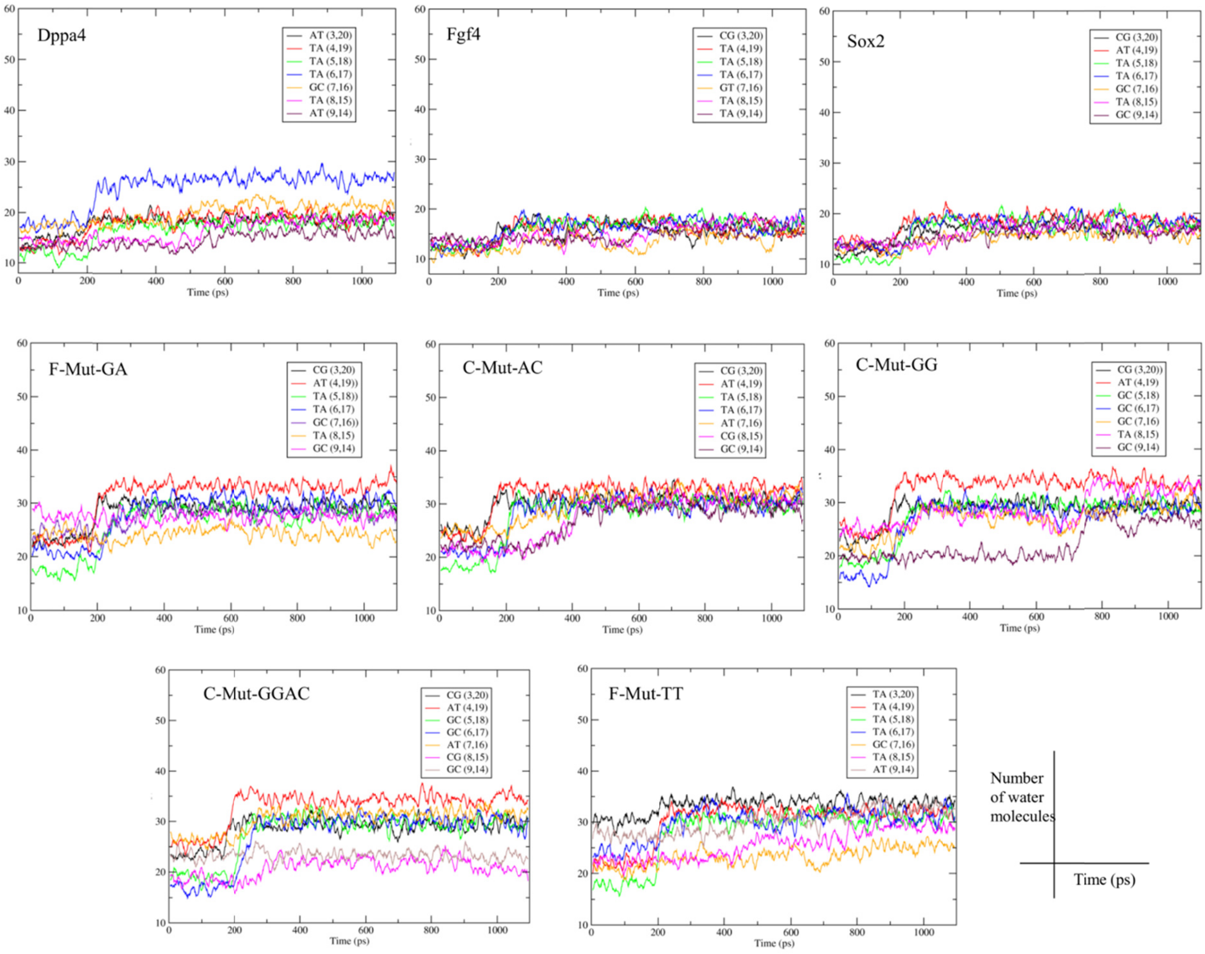

Fig. S5. Water-mediated interaction with bps of DNA. Interactions of the water molecules with DNA bps during the process of dissociation of Sox2 from its target DNA. The increase in the number of water molecules show the ability of each bp of highly negatively charged DNA to attract more water molecules during the process of dissociation. The bps of the mutant complexes show an increased number of water molecule interactions than the experimental complexes.

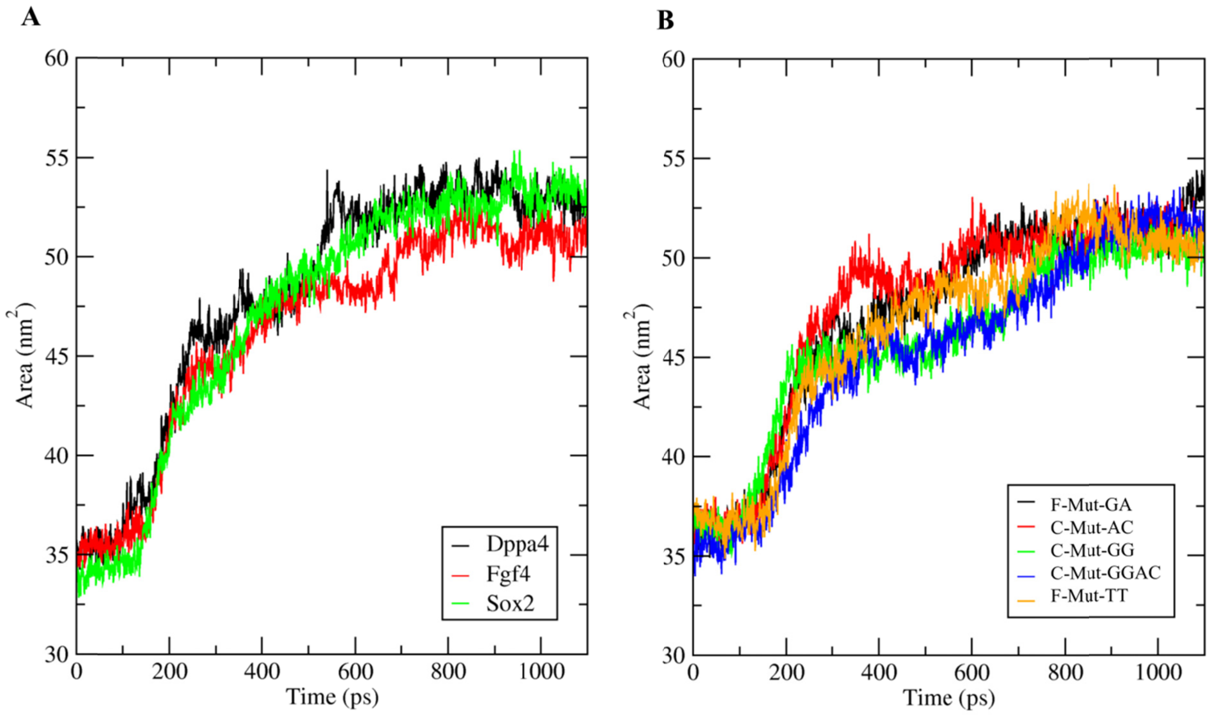

Fig. S6. Comparison of the solvent-accessible surface areas (SASAs) during dissociation. (A) A SASA graph for the positive-control complexes showing an increase in the SASA with a decrease in the electrostatic interaction when Sox2 moves away from the DNA. (B) A SASA graph for the mutant complexes showing an increase in the SASA with a decrease in the electrostatic interaction as Sox2 moves away from the DNA.

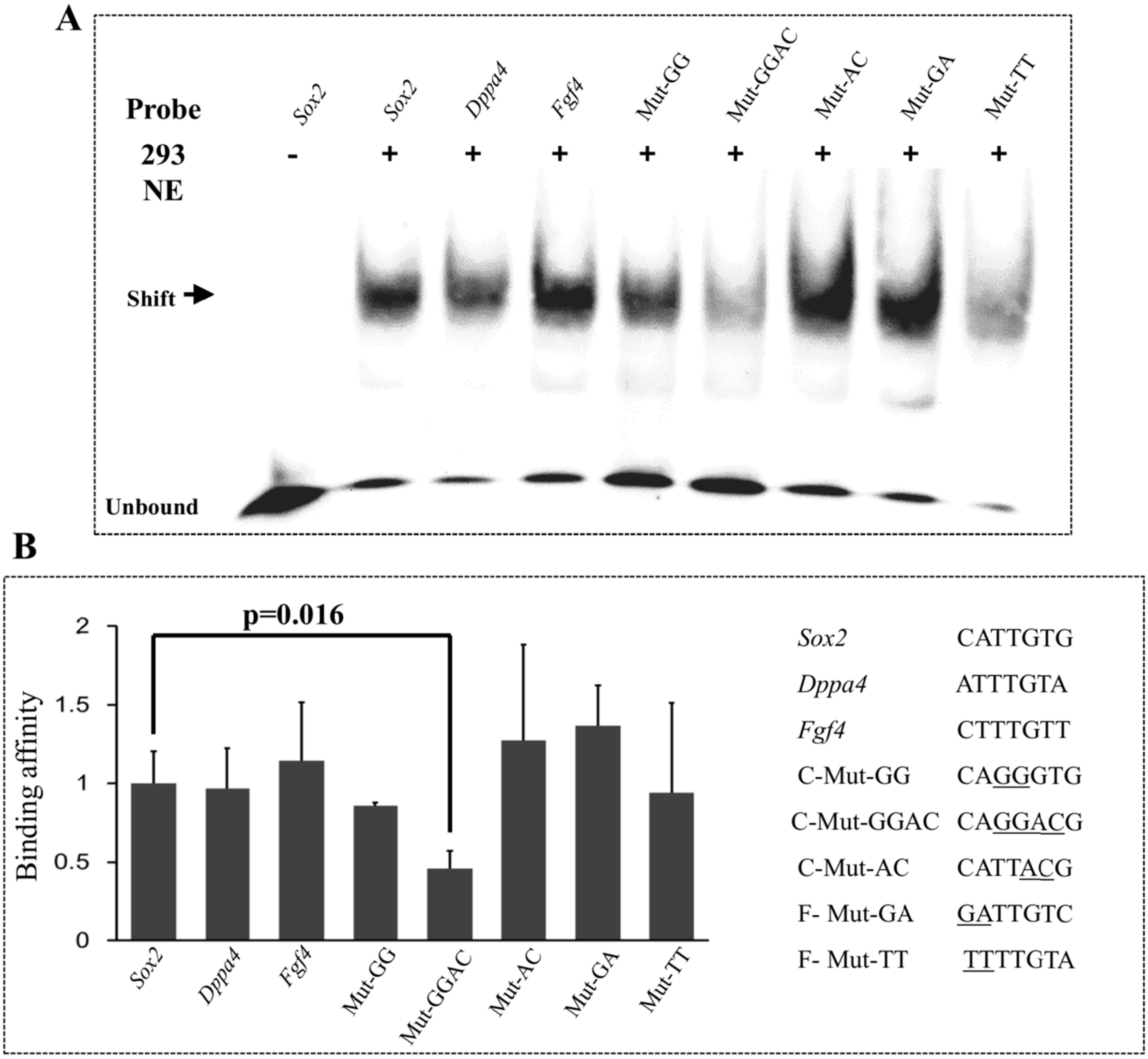

Fig. S7. EMSA experiment intended to determine binding affinity. (A) EMSAs comparing Sox2's binding affinity for different binding motifs in DNA. F-Mut-GA and C-Mut-AC have better affinity than the experimentally validated binding patterns of Sox2. The probes are biotin labeled. The representative gel shown was obtained after scanning on a LAS-1000 system (FUJIFILM). (B) A histogram of each complex showing quantitative determination of Sox2-binding patterns. Error bars represent standard deviations, and the statistical significance of *Sox2* and C-Mut-GGAC is shown according to Student's *t* test.

Protocol for Electrophoresis Mobility Shift Assay (EMSA)

Cell culture

The cell line HEK293 was purchased from the American Type Culture Collection (ATCC # CRL-1573; Manassas, VA, USA). The cells were grown in high-glucose Dulbecco's Modified Eagle's Medium supplemented with fetal bovine serum (GE Healthcare, Logan, UT, USA) and maintained at 37°C and 5% CO_2.

Electrophoresis Mobility Shift Assay (EMSA)

HEK293 nuclear extract (293-NE) was prepared using NE-PER™ Nuclear and Cytoplasmic Extraction Reagents (Thermo Scientific, Waltham, MA, USA) supplemented with the Halt protease inhibitor cocktail (Thermo Scientific). Oligonucleotide probes used in the EMSA are listed in Table S1. For each reaction, 20 fmol of a 5′-biotin-labeled double-stranded probe was incubated for 2 hours on ice with 293-NE in a 20 µl reaction buffer consisting of 10 mM Tris-HCl pH 8.0, 50 µM $ZnCl_2$, 150 mM KCl, 1 mM DTT, 10% glycerol, 0.1 mg/ml BSA, 0.1% NP-40, and 1 µg/µl poly-dIdC. Reactions were shifted on a 6% 0.5× TBE polyacrylamide gel at 4°C, which was dried before exposure of autoradiography films, which were analyzed on a LAS-1000 system (FUJIFILM, Tokyo, Japan). Quantitative analysis of DNA-binding affinity was performed using the GelQuant.NET 1.8.2 software. Student's *t* test was applied to measure the significance, and data with $p < 0.05$ were considered significant. The assay was performed for five times, and three runs were taken for the calculations.

Table S1. A list of EMSA oligonucleotide sequences. The binding patterns of Sox2 are underlined, and the mutant sites are indicated in red. The 5′ and 3′ ends of the DNA strands are labeled.

No	Complex name	DNA sequence
1	*Sox2*	5' AGGTACCTATT **CATTGTG** ATGCAAATAAGCTTC 3'
2	*Dppa4*	5' AGGTACCTATT **ATTTGTA** ATGCAAATAAGCTTC 3'
3	*Fgf4*	5' AGGTACCTATT **CTTTGTT** ATGCAAATAAGCTTC 3'
4	C-Mut-GG	5' AGGTACCTATT **CAGGGTG** ATGCAAATAAGCTTC 3'
5	C-Mut-GGAC	5' AGGTACCTATT **CAGGACG** ATGCAAATAAGCTTC 3'
6	C-Mut-AC	5' AGGTACCTATT **CATTACG** ATGCAAATAAGCTTC 3'
7	F-Mut-GA	5' AGGTACCTATT **GATTGTC** ATGCAAATAAGCTTC 3'
8	F-Mut-TT	5' AGGTACCTATT **TTTTGTA** ATGCAAATAAGCTTC 3'

Supplementary Movies: Demonstration of a simulated dissociation pathway for the complexes under study. Movies showing the process of dissociation of Sox2 from DNA (major dissociation, N-terminal, and C-terminal dissociation) and the structural changes during the steered molecular dynamics simulation. The DNA is orange, Sox2 protein is cyan, and the C-terminal charged residues are green. The movies were created using Chimera.

Movie S1. Dissociation of Sox2 from DNA in the *Sox2* promoter (CATTGTG).

Movie S2. Dissociation of Sox2 from DNA in the *Fgf4* promoter (CTTTGTT).

Movie S3. Dissociation of Sox2 from DNA in the *Dppa4* promoter (ATTTGTA).

Movie S4. Dissociation of Sox2 from DNA in F-Mut-TT (TTTTGTA).

Movie S5. Dissociation of Sox2 from DNA in F-Mut-GA with (GATTGTC).

Movie S6. Dissociation of Sox2 from DNA in C-Mut-AC (CATTACG).

Movie S7. Dissociation of Sox2 from DNA in C-Mut-GG (CAGGGTG).

Movie S8. Dissociation of Sox2 from DNA in C-Mut-GGAC (CAGGACG).

Movie S9. Dissociation of Sox2 from C-Mut-GGAC (CAGGACG) with a force constant of 50 kcal/(mol·nm^2).

Supplementary File S1. Sox2 binding patterns matching with human ESC target genes.

Supplementary File S2. Sox2 binding patterns matching with mouse ESC target genes.
